# Go big or go home: A model-based assessment of general strategies to slow the spread of forest pests via infested firewood

**DOI:** 10.1371/journal.pone.0238979

**Published:** 2020-09-15

**Authors:** Peter C. Jentsch, Chris T. Bauch, Denys Yemshanov, Madhur Anand

**Affiliations:** 1 Department of Applied Mathematics, University of Waterloo, Waterloo, Ontario, Canada; 2 Natural Resources Canada, Canadian Forest Service, Great Lakes Forestry Centre, Sault Ste. Marie, Ontario, Canada; 3 School of Environmental Sciences, University of Guelph, Guelph, Ontario, Canada; University of Pavia, ITALY

## Abstract

Invasive pests, such as emerald ash borer or Asian longhorn beetle, have been responsible for unprecedented ecological and economic damage in eastern North America. These and other wood-boring invasive insects can spread to new areas through human transport of untreated firewood. Behaviour, such as transport of firewood, is affected not only by immediate material benefits and costs, but also by social forces. Potential approaches to reduce the spread of wood-boring pests through firewood include raising awareness of the problem and increasing the social costs of the damages incurred by transporting firewood. In order to evaluate the efficacy of these measures, we create a coupled social-ecological model of firewood transport, pest spread, and social dynamics, on a geographical network of camper travel between recreational destinations. We also evaluate interventions aimed to slow the spread of invasive pests with untreated firewood, such as inspections at checkpoints to stop the movement of transported firewood and quarantine of high-risk locations. We find that public information and awareness programs can be effective only if the rate of spread of the pest between and within forested areas is slow. Direct intervention via inspections at checkpoints can only be successful if a high proportion of the infested firewood is intercepted. Patch quarantine is only effective if sufficiently many locations can be included in the quarantine and if the quarantine begins early. Our results indicate that the current, relatively low levels of public outreach activities and lack of adequate funding are likely to render inspections, quarantine and public outreach efforts ineffective.

## Introduction

Invasive species pose a significant economic and ecological threat to Canada’s forest ecosystems [1, 2]. In North America, significant funding has been allocated by federal, state and provincial agencies for large-scale control programs to prevent or mitigate these damages with mixed success [3, 4]. Controlling the spread of invasive pests can be difficult because the long-distance spread of invasive organisms is often assisted by human activities [1, 5]. For example, introduction and spread of Emerald ash borer, a harmful forest pest in the North America [6–8] has been attributed to human factors, such as vehicle transport [9] and recreational travel [10].

The growing problem of invasive species is broadly associated with human mobility, including recreational travel [1, 5, 11, 12]. Outdoor recreation is widespread in North America, and the extent of recreational activities is expected to increase [13–15]. In North America, national, provincial and state parks, national forests, and state and Crown lands are common destinations for recreational activities [16, 17]. In Canada, recreational activities, especially camper travel, often take place in forested areas and may contribute to spread of harmful invasive pests. In particular, the movement of untreated firewood by campers has been widely acknowledged as a potential introduction pathway for invasive forest pests [2, 10, 18–20]. Movement of untreated firewood has been linked to the spread of two harmful wood-boring pests, the Asian longhorned beetle (*Anoplophora glabripennis* Motschulsky) and the emerald ash borer (*Agrilus plannipennis* Fairmaire), in the United States and Canada (USaC) [21, 22].

Firewood is often moved to distant locations by campers for recreational purposes [2, 23]. For example, Haack et al. (2010) has found live bark- and wood-boring insects in 23% of the firewood pieces, surrendered at the checkpoint station at Mackinac bridge connecting Michigan’s Lower and Upper Peninsulas and an additional 41% had signs of prior borer infestation. Jacobi et al. (2011) reported the emergence of live insects from 47% of the firewood bundles purchased from various US retailers. To reduce the risk of future pest infestations, USaC have implemented various regulations on movement of untreated firewood, including bans for out-of-province movement of untreated firewood and restrictions for its transport by short distances [19, 23–25]. Also a number of public outreach campaigns have been undertaken to educate the general public about the threats associated with the movement of untreated firewood and its potential to spread harmful invasive pests. Several strategies have been developed to prevent (or minimize) the movement of firewood with recreational travel, including outreach campaigns in public media, enforcements with the inspections at check points for transported firewood, and area quarantine with the restrictions on firewood movement from/to the area of concern. In particular, public outreach campaigns have become widespread with significant funding by local, municipal, and provincial governments on measures such as advertisements along major highways and in public media and educational information in websites and printed media. The use of enforcement and quarantine options is less common but is gaining acceptance as a last resort measure and was implemented at least a few times over the past decade, to varying degrees of success [10, 18, 25].

Assessing the efficacy of the measures aimed to prevent the movement of firewood with recreational travel is a daunting task. Outreach campaigns may spread information widely but there is no guarantee that campers will pay attention and comply with the firewood restriction warnings. Many outreach activities (such as posting ads in public media or distributing flyers) are often implemented sporadically at local scales using local municipal and provincial budgets [19], which makes the assessment of their efficiency difficult. These activities may simultaneously occur in different places and times with little or no coordination, and are difficult to track in time and space.

Alternatively, the enforcement options (such as quarantine or checkpoint inspections for illegal movement of firewood) are gaining acceptance and may be perceived as more effective localized means to stop the movement of untreated firewood by campers. Nevertheless, assessing the effectiveness of enforcement actions is challenging due to a very small scale of enforcement actions (often implemented by individual states or provinces at selected locations) and lack of compliance data.

Mechanistic models of forest invasions have been studied for decades [26, 27], but explicit modelling and consideration of human factors, and the feedback between humans and the environment is relatively new. Ali et al. and Barlow et al. [18, 28] proposed two models of forest pest spread through firewood transport. The first study presented a differential equation model, and the second an agent-based model, both assuming that humans are the primary long-distance movers of forest pests. The models proposed in [18, 28] coupled infestation dynamics with the social dynamics. However both studies considered a small and idealized spatial structure: two patches in Barlow’s et al. [18] study and ten patches in Ali‘s et al. model [28]. Often, illegal movement of firewood occurs over large distances and may involve visits to multiple recreational destinations that are connected differently to one another.

In this study we consider movement of infested firewood to multiple recreational destinations over a complex recreational travel network. We explore the efficacy of common measures aimed to stop the movement of untreated firewood by recreational travelers. To accomplish this, we propose a mechanistic differential equation model that combines human-mediated movement of forest pests through a camper travel network that includes nonlinear feedbacks from social factors, such as population response to strategies preventing the movement of untreated firewood. We identify three basic methods to stop or slow the spread of invasive pests by transport of infested firewood: public awareness campaigns, direct interception of transported firewood at checkpoints near recreational destinations, and quarantining recreational destination sites for movement of firewood. While the first option is more common, the latter has been implemented seldom over the past decade due to legal and liability constraints [24, 29–31]. We implement the options for intercepting the movement of firewood to slow the spread of invasive pests in a mechanistic metapopulation model, and use the replicator equation to represent social learning dynamics (see [18, 32–34]). We also evaluate local quarantine at recreational destinations as an alternative control method. Quarantine means closing the site to visitors for a length of time, in order to reduce the amount of transported firewood and slow spread of invasive organisms from other infested locations. Our implementation of quarantine measures follow common practices aimed to slow the spread of invasive species (such as the spread of emerald ash borer in USaC [35, 36]). We apply our mechanistic model to explore the effectiveness of these control measures to slow the spread of an idealized wood-boring invasive pest moved to a set of recreational destinations by recreational travelers transporting untreated firewood. We apply the model to a network of provincial parks and campgrounds in three provinces of central Canada—Manitoba, Ontario, and Quebec.

## Materials and methods

We consider a landscape of *N* patches, where a patch is represented as *i* ∈ [1, *N*]. Each patch represents a recreational destination (eg. provincial parks and campgrounds) with associated neighbouring human population centres. Each patch undergoes its own internal pest and social dynamics. We describe the spread of an invasive pest with the movement of firewood through the network of N patches with a mechanistic metapopulation model based on [18] that captures the spread of an infestation between the patches. The advantage to metapopulation models in this context is suitability for capturing dynamics of a highly fragmented population spread over a broad geographic region. Using the data documenting reservations of provincial campgrounds in Ontario, Manitoba and Quebec ([37], we created a graph of camper travels which depicts a spatial travel network between origin locations (which correspond to residential addresses of camper travelers) and recreational destinations (campgrounds in provincial parks and historic sites). The camper travel network is described by a graph with coefficients *P*_*i*,*j*_ denoting the relative frequency of camper movements between origin locations *j* and recreational destination locations *i* (see more details on spatial data in section). Specifically, for a given location *j*, *P*_*i*,*j*_ is the fraction of trips that go from *j* to *i* each year, so we have $\sum_{i=1}^{N}P_{i,j}=1$. Consider a patch *i* with an enforcement intervention, such as firewood movement quarantine, or a voluntary firewood surrender checkpoint aimed to stop the flow of untreated firewood from that location. We denote *C*_*e*_ as the percentage of infested firewood that can be intercepted on a route between two locations *i* and *j*, 0 ≤ *C*_*e*_ ≤ 1. Interception at *i* may reduce the movement of infested firewood from a patch *i* to other patches *j*, so *C*_*e*_ indicates, in relative terms, the magnitude of interception efforts.

We also consider a public outreach campaign that can take place at a patch *i*. It is common that only a portion of campers visiting a patch *i* may be aware of and decide to comply with the public outreach message. We model the social awareness campaign as an increase of the net social cost of transporting firewood. We further conduct sensitivity analyses to compare the efficacy of enforcement vs. outreach measures aimed to stop the movement of firewood and reduce the rates of infestation.

### Pest spread model

We begin with defining the equation for a population of susceptible host trees that may be attacked by an invasive pest. The pest can be introduced though untreated infested firewood. Variables, their interpretations, and corresponding baseline ranges are shown in Table 1. We assume that a tree population that is susceptible to pest attack undergoes logistic growth in the absence of infestation to a carrying capacity *K*. The population of susceptible trees, *S*_*i*_(*t*), at a patch *i* is being infested from firewood arriving with campers at *i* at a rate *A*:

$$\begin{array}{c} \frac{dS_{i}}{dt}=\underset{\text{Logistic}\;\text{Growth}\;\text{Of}\;\text{Forest}}{\underset{︸}{rS_{i}(1-\frac{\left(S_{i}+I_{i}\right)}{K})}}-\underset{\text{Infestation}\;\text{term}}{\underset{︸}{AS_{i}\left(I_{i}+B_{i}\right)\theta_{k}\left(I_{i}-I_{a}\right)}} \end{array}$$ (1)

where *θ*_*k*_(*I*_*i*_) is a sigmoid function such as

$$\begin{array}{c} \theta_{k}\left(x\right)=\frac{1}{1+e^{-kx}} \end{array}$$ (2)

Terms *S*_*i*_ and *I*_*i*_ are the number of susceptible and infected trees, respectively, at patch *i*. *B*_*i*_ is the quantity of infested firewood in patch *i*, which we assume has the same probability of pest transmission within patch as infested trees. We choose the carrying capacity *K* to be the same in each patch for simplicity. The term *AS*_*i*_*I*_*i*_*θ*_*k*_(*I*_*i*_ − *I*_*a*_) represents intra-patch infestation with a density dependent population, parameterized by *k* and *I*_*a*_, where *I*_*a*_ determines population of infested trees at which transmission is halved, and *k* is is a constant which affects the sharpness of the transition of *θ*_*k*_(*x*) at *I*_*a*_. We assume that there is an influx of pest organisms entering a patch *i* with firewood which defines the propagule pressure at *i*. Infested trees at *i* are assumed to die at a constant rate *γ*, giving the following equation for the infested tree population of a patch.

$$\begin{array}{c} \frac{dI_{i}}{dt}=\underset{\text{Death}\;\text{of}\;\text{infested}\;\text{trees}}{\underset{︸}{-\gamma I_{i}}}+\underset{\text{Susceptible}\;\text{become}\;\text{infested}}{\underset{︸}{AS_{i}\left(I_{i}+B_{i}\right)\theta_{k}\left(I_{i}-I_{a}\right)}} \end{array}$$ (3)

The patches are spatially coupled through the transport of firewood by recreational travelers. The infestation rate at *i* depends on the number of visitors transporting infested firewood to *i*, which is also a function of the social dynamics at *i*, such as the enforcement, or public outreach measures described by a utility function, presented in [33], and applied to forest modelling in [18, 38]. Let *L*_*i*_ be the proportion of visitors to patch *i* who do not transport firewood and buy it locally, and *d* rate of exportation of infested logs. The rate of infested wood coming into patch *i* can be estimated as:

$$\begin{array}{c} d\sum_{j=1,j\neq i}^{N}P_{i,j}\left(1-C_{e}\right)\left(1-L_{j}\right)I_{j} \end{array}$$

The dynamics of *L*_*i*_ (the number of local transporters in patch *i*), is modelled by a replicator dynamics model that is suitable for describing systems where social learning occurs [33, 34], and is described in the section below.

**Table 1:** Parameters and default values.

Name	Default Value, (Range explored)	Units	Interpretation
*N*	2250	Patches	Number of patches in the network
*S*_*i*_	Site specific	Trees	Number of susceptible trees in patch *i*
*I*_*i*_	Site specific	Trees	Number of infested trees in patch *i*
*B*_*i*_	Site specific	Trees	Infested firewood in patch *i*
*L*_*i*_	Site specific	Unitless	Fraction of local strategists in patch *i*
*r*	0.02, [0.01, 0.06]	New trees per tree per year	Tree growth rate
*A*	0.001, [0.00065, 0.0014]	Number of infested trees per susceptible-infested contact per year	Transmission rate of pest
*γ*	1.4, [0.8, 1.8]	Trees per year	Decay rate for infested trees
*K*	5000	Trees	Carrying capacity of each patch
*U*	0, [-5, 5]	Utility	Social cost to transport firewood, or incentive to buy locally
*C*_*e*_	0, [0.0, 1.0]	Unitless	Interception fraction
*f*	0.1, [0.01, 0.13]	Utility per capita	Impact of local infection on strategy
*s*	0.1	Utility per capita	Strength of social norms
*σ*	0.1	Strategy changes per capita per year	Rate of social learning
*P*_*i*,*j*_	See below	Unitless	Fraction of trips that go from *j* to *i* each year.
*d*	0.1 [0.05, 0.3]	Logs per year	Rate of transmission of infested firewood between patches
*I*_*a*_	1 [0.5, 5]	Trees	Value at which transmission rate of pest is halved due to density dependence
*k*	1	Unitless	Steepness of sigmoid function
*V*	Empty, [0 patches, 500 patches]	Patches	Set of patches to be quarantined
Δ*t*	0, [0, 5]	Years	Length of quarantine
*t*_0_	0, [0, 5]	Years	Time between initial infestation and patch quarantine

### Social dynamics model

We model the proportion of visitors who choose to use local firewood, *L*_*i*_ as a function of both the perceived threat of introduced pests, and the social cost of illegally transporting infested firewood. We refer to visitors who choose to use local firewood as local strategists, and visitors who do not use local firewood as transport strategists hereafter. Let *C*_*t*_ be the cost of transporting firewood and *C*_*l*_ the cost to obtain it locally (and therefore avoid moving invasive pests to a patch *i*). We adopt the social influence model from [18], which is based on models of [33] and [34], which we will summarize below. We define the social utilities corresponding to the strategies of transporting firewood (*P*_*t*_) and buying it locally (*P*_*l*_) as

$$\begin{array}{c} P_{t}=-C_{t}+s\left(0.5-L_{i}\right)-fI_{i} \end{array}$$

$$\begin{array}{c} P_{l}=-C_{l}+s\left(L_{i}-0.5\right) \end{array}$$

Transportation becomes a less attractive strategy if infestation is more prevalent, depending on the size of *f*. The parameter *f* controls the extent to which a local infestation causes behaviour change in that population. The parameter *s* controls the degree to which individuals are influenced by the the majority opinion in their patch (i.e. peer pressure). We assume that both local strategists and transport strategists in a patch *i*, given by *L*_*i*_ and 1 − *L*_*i*_ respectively, decide whether to change their strategy at the same rate, *σ*. Their decision is made by considering which strategy will maximize their utility *P*_*l*_ − *P*_*t*_ at that point, leading to the following expression for the rate of change of the local strategist population:

$$\begin{array}{c} \frac{dL_{i}}{dt}=\sigma L_{i}\left(1-L_{i}\right)\left(P_{l}-P_{t}\right) \end{array}$$

We replace the individual costs of *C*_*t*_, *C*_*l*_ with the net utility value *U* = *C*_*t*_ − *C*_*l*_. The cost difference *U* abstracts from the explicit definition of costs of using firewood [18] and allows including exogenous social incentives and motivation, such as awareness about the problem or any other form of social influence from outside each location *i*. A term *B*_*i*_ is introduced to represent the amount of local firewood available in patch *i*. For simplicity, we assume that the tree mortality rate at a patch *i* is only caused by infestation, so the mortality rate is the same as the death rate of the infested trees

$$\begin{array}{c} \frac{dB_{i}}{dt}=\underset{\text{Decay}\;\text{of}\;\text{fallen}\;\text{wood}}{\underset{︸}{-\gamma B_{i}}}+\underset{\text{Import}\;\text{of}\;\text{fallen}\;\text{wood}}{\underset{︸}{d\sum_{j=1,j\neq i}^{N}P_{i,j}\left(1-C_{e}\right)\left(1-L_{j}\right)I_{j}}} \end{array}$$ (4)

Because the infested wood imported into patch *i* in Eq 4 must come from another patch in the system, we subtract the corresponding term for leaving wood, $d\sum_{j=1,j\neq i}^{N}P_{j,i}\left(1-C_{e}\right)\left(1-L_{i}\right)I_{i}$ from Eq 6 which describes the rate of change of infested population in a patch *i*. Using the notation in Eqs (5), (6), (7) and (8), we formulate the problem of buying firewood locally vs. transporting it from other potentially infested locations as follows:

$$\begin{array}{c} \frac{dS_{i}}{dt}=\underset{\text{Logistic}\;\text{Growth}\;\text{Of}\;\text{Forest}}{\underset{︸}{rS_{i}(1-\frac{\left(S_{i}+I_{i}\right)}{K})}}-\underset{\text{Infestation}\;\text{term}}{\underset{︸}{AS_{i}\left(I_{i}+B_{i}\right)\theta_{k}\left(I_{i}-I_{a}\right)}} \end{array}$$ (5)

$$\begin{array}{c} \frac{dI_{i}}{dt}=\underset{\text{Death}\;\text{of}\;\text{infested}\;\text{trees}}{\underset{︸}{-\gamma I_{i}}}+\underset{\text{Susceptibles}\;\text{become}\;\text{infested}}{\underset{︸}{AS_{i}\left(I_{i}+B_{i}\right)\theta_{k}\left(I_{i}-I_{a}\right)}}-\underset{\text{Total}\;\text{infested}\;\text{wood}\;\text{leaving}\;\text{due}\;\text{to}\;\text{transport}}{\underset{︸}{d\sum_{j=1,j\neq i}^{N}P_{j,i}\left(1-C_{e}\right)\left(1-L_{i}\right)I_{i}}} \end{array}$$ (6)

$$\begin{array}{c} \frac{dB_{i}}{dt}=\underset{\text{Decay}\;\text{of}\;\text{firewood}}{\underset{︸}{-\gamma B_{i}}}+\underset{\text{Import}\;\text{of}\;\text{fallen}\;\text{wood}}{\underset{︸}{d\sum_{j=1,j\neq i}^{N}P_{i,j}\left(1-C_{e}\right)\left(1-L_{j}\right)I_{j}}} \end{array}$$ (7)

$$\begin{array}{c} \frac{dL_{i}}{dt}=\sigma L_{i}\left(1-L_{i}\right)\left(\underset{\text{Net}\;\text{cost}\;\text{to}\;\text{transport}\;\text{firewood}}{\underset{︸}{U}}+\underset{\text{Social}\;\text{influence}\;\text{term}}{\underset{︸}{s(2L_{i}-1)}}+\underset{\text{Impact}\;\text{of}\;\text{infestation}}{\underset{︸}{fI_{i}}}\right) \end{array}$$ (8)

Table 1 lists the model notation.

### Patch-quarantine strategies

Let *V* ⊂ [1, *N*] be a set of patches under a quarantine. We use the patches (nodes of the camper travel network) with the largest (shortest-path) betweenness centrality [39, 40], which is a common approach for selecting quarantine nodes in vaccination studies [41]. Betweenness centrality measures the extent to which a node lies on paths between other nodes and is used to detect the amount of influence a particular node has over the flow of information in a graph. The measure is often used to find nodes that serve as critical links between different parts of a graph. Formally, the shortest-path betweenness centrality of a node *i* ∈ *V* on a weighted graph *G* is

$$\begin{array}{c} g\left(i\right)=\sum_{i\neq s\neq t;s,t\in G}\frac{g_{st}\left(i\right)}{g_{st}} \end{array}$$

where *g*_*st*_ is the number of shortest paths between nodes *s*, *t* and *g*_*st*_(*i*) is the number of geodesic paths between nodes *s*, *t* that go through node *i*. Both of these measurements calculate path length with respect to the weights of G,. In words, the betweenness centrality *g*(*i*) of a node *i* is the probability that *i* lies on a shortest path between some two nodes in *G*. In our camper travel network, higher weights denote more frequent trips, so for the purposes of determining the betweenness centrality, the weight of each edge (*i*, *j*) is *max*_*i*,*j*_(*P*_*ij*_) + 1 − *P*_*ij*_.

We model the implementation of firewood quarantine strategies at patches *V* by introducing a time-dependent term in Eqs (6) and (7). Let *t*_0_, and Δ*t* be the starting time of the quarantine and the length of the quarantine respectively. Let *H*_*c*_(*x*, Δ*t*), defined as

$$\begin{array}{c} H_{c}\left(x,\Delta t\right)=\{\begin{array}{cc} 1 & x<0\\ 0 & 0\leq x\leq \Delta t\\ 1 & x>\Delta t \end{array} \end{array}$$

be an upside-down boxcar function of length Δ*t*. This function acts as a switch which is “off” whenever *x* ∈ [0, Δ*t*]. With this function, we can modify Eqs (6) and (7) so that patches *i* ∈ *V* do not import or export firewood whenever *x* ∈ [0, Δ*t*].

If *i* ∈ *V*,

$$\begin{array}{c} \frac{dI_{i}}{dt}=-\gamma I_{i}+AS_{i}\left(I_{i}+B_{i}\right)\theta_{k}\left(I_{i}+B_{i}\right)-dH_{c}\left(t-t_{0},\Delta t\right)\sum_{j=1,j\neq i}^{N}P_{j,i}\left(1-C_{e}\right)\left(1-L_{i}\right)I_{i} \end{array}$$ (9)

$$\begin{array}{c} \frac{dB_{i}}{dt}=-\gamma B_{i}+dH_{c}\left(t-t_{0},\Delta t\right)\sum_{j=1,j\neq i}^{N}P_{i,j}\left(1-C_{e}\right)\left(1-L_{j}\right)I_{j} \end{array}$$ (10)

Note that the only difference in the new Eqs (9) and (10) from Eqs (6) and (7) is in the last term denoting the interactions with neighbouring nodes. The equations for patches not in under quarantine (i.e., not in *V*) require us to distinguish arcs that connect to and from nodes under quarantine in *V*.

If *i* ∉ *V*,

$$\begin{array}{cc} \frac{dI_{i}}{dt}=-\gamma I_{i}+AS_{i}\left(I_{i}+B_{i}\right)\theta_{k}\left(I_{i}+B_{i}\right) & -\sum_{j=1,j\neq i,j\notin V}^{N}P_{j,i}\left(1-C_{e}\right)\left(1-L_{i}\right)I_{i}\\ & -dH_{c}\left(t-t_{0},\Delta t\right)\sum_{j=1,j\neq i,j\in V}^{N}P_{j,i}\left(1-C_{e}\right)\left(1-L_{i}\right)I_{i} \end{array}$$ (11)

$$\begin{array}{c} \frac{dB_{i}}{dt}=-\gamma B_{i}+\sum_{j=1,j\neq i,j\notin V}^{N}dP_{i,j}\left(1-C_{e}\right)\left(1-L_{j}\right)I_{j}+dH_{c}\left(t-t_{0},\Delta t\right)\sum_{j=1,j\neq i,j\in V}^{N}P_{i,j}\left(1-C_{e}\right)\left(1-L_{j}\right)I_{j} \end{array}$$ (12)

In Eqs (11) and (12) we split the summation term into two summations, one over all patches which are not under quarantine (i.e., not in the set *V*) and patches under quarantine in *V*. The latter summation is multiplied by a term, *H*_*c*_(*t*−*t*_0_, Δ*t*) which switches on and off the quarantine conditions.

### Parameterization

**Fig 1:**
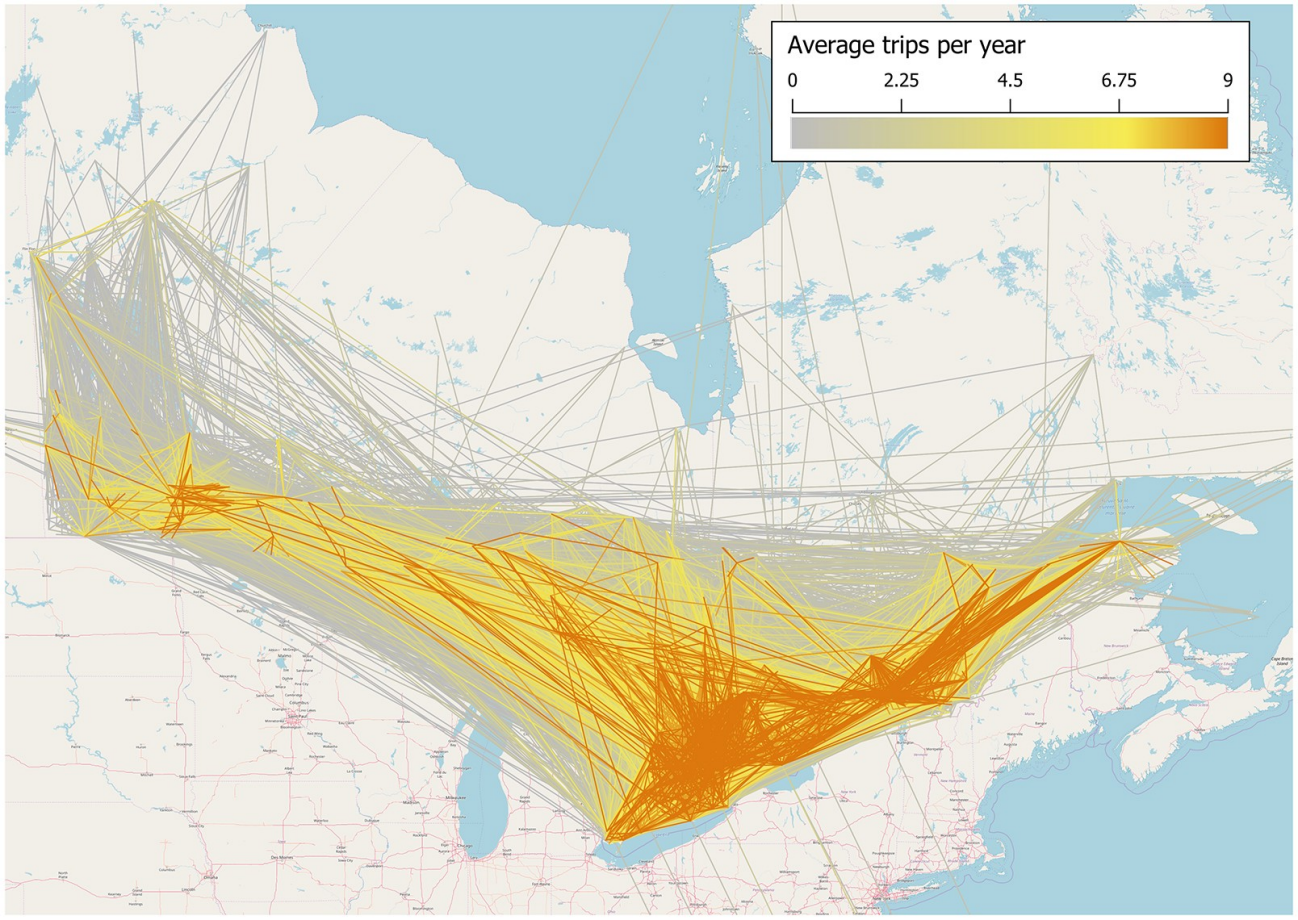
Camper travel network in Ontario, Quebec and Manitoba. Darker (more orange) lines represent more trips.

We used data from [10] and [37], to quantify the risk of firewood transport to recreational destinations in Central Canada. The data documented the movements of campers to provincial campgrounds in Ontario, Quebec and Manitoba. Such data are maintained by provincial ministries of natural resources (MNRs). The dataset included a large number of potential origin sites (i.e., approximately 9000 locations). To reduce the computational burden, we reduced the size of the camper travel network by including all recreational destination locations but considering only the origin locations in the Canadian provinces of Ontario, Manitoba, and Quebec. We further reduced the size of the network by selecting most travelled routes. We selected the largest subgraph with a minimum degree of 10 (the 10-core of the graph) which considered only the most connected nodes, with largest impact on pest transmission. We implemented the procedure using the NetworkX library [42]. The final camper travel network included 2250 sites (Fig 1).

Because the model uses a large camper travel network it has a very large parameter space, and many of the parameters, especially those in Eq 8, are difficult to estimate directly from data. In this study we are exploring the region of parameter space that most closely approximates the dynamics in real infestations, such as the typical size and duration of the recent emerald ash borer outbreak in eastern Canada. To select the most relevant range of the social influence parameters, *σ*, *s*, *f*, which are difficult to estimate from the literature, we did sensitivity analyses over a wide range of these parameters, and identified the parameter space where these parameters had the largest effect on the model dynamics, and where the course of the invasion was realistic. The inter-patch and intra-patch infection rate parameters, *d*, *A*, were selected to infest and eventually kill at least 95% of the tree population within 10 to 15 years.

We integrated Eqs (5)–(8) using code written in the Julia language, using the JuliaDiffEq library [43]. The integration was run on the Compute Canada clusters. Our primary focus was to explore the relative impacts of firewood enforcement versus public outreach and their abilities to reduce pest infestation rates across the camper travel network. We consider a hypothetical scenario where a harmful invasive pest is introduced in the largest urban center in eastern Canada with foreign imports (Greater Toronto Area, GTA) and assume that the bulk host tree population in the GTA is infested. This scenario is based on a history of past entries of invasive wood-boring pests to the GTA with foreign imports (such as introduction of Asian longhorned beetle in Toronto and Mississauga [44]).

### Assessing intervention efficacy

The primary statistic we use to assess the total mortality of an infestation after *t* years is the average cumulative infested population, $\frac{1}{N}\sum_{i=1}^{N}T_{i}\left(t\right)$. To calculate *T*_*i*_(*t*), the cumulative infested population at patch *i* and time *t*, we solve the following equation in addition to the model equations.

$$\begin{array}{c} \frac{dT_{i}}{dt}=AS_{i}\left(I_{i}+B_{i}\right)\theta_{k}\left(I_{i}-I_{a}\right) \end{array}$$ (13)

The right-hand side of Eq 13 is the only positive term of Eq 6, so it increases when new infested trees are added to *I*_*i*_(*t*), but does not decrease when infested trees die, thereby counting the total number of infestations.

Since it is difficult to determine what utility value *U*, which defines the social cost of transport, corresponds to the current level of funding, we try to answer whether it would be beneficial to increase the funding, which we call the marginal benefit of increasing *U*. Given a time $\bar{t}$, we calculate $T(\bar{t})$ for a set of *U* ∈ [−5, 5], then we fit a linear function of *U* to these points. We find a first-order approximation of $T(\bar{t})$ change per unit *U* (Fig 4) for a given set of parameters and time $\bar{t}$. A positive slope indicates that total infested tree population increases when *U* is increased, which means that increasing *U* does not reduce the impact of the pest (at least, to a first approximation). In Figs (4)–(6) this method is used to show how the total number of infested trees changes with respect to an increase in *U*, as a function of parameters and time.

## Results

**Fig 2:**
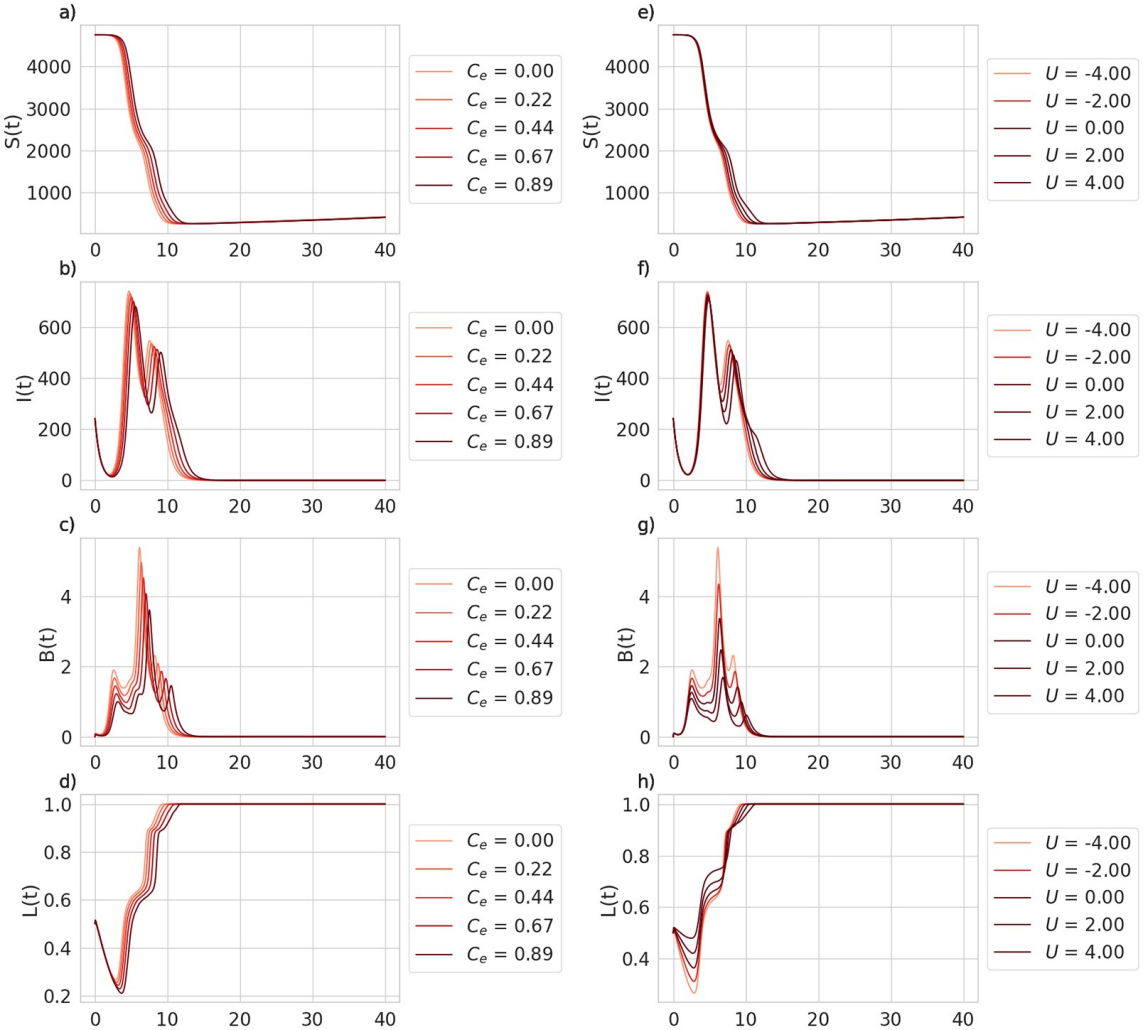
Time series of model variables as a function of interventions, direct (raising *C*_*e*_, panels a—d) and through social pressure (raising *U*, panels e—h). The former intervention, panels a-d, means an increase of social pressure on people who choose to transport firewood (i.e. increasing the *U* value), and the latter refers to direct interception of firewood (i.e. increasing the *C*_*e*_ value). Terms $L\left(t\right)=\frac{1}{N}\sum_{i=1}^{N}S_{i}\left(t\right)$, $I\left(t\right)=\frac{1}{N}\sum_{i=1}^{N}I_{i}\left(t\right)$, $B\left(t\right)=\frac{1}{N}\sum_{i=1}^{N}B_{i}\left(t\right)$, $L\left(t\right)=\frac{1}{N}\sum_{i=1}^{N}L_{i}\left(t\right)$ are the averages of the state variables over all patches. *S*(*t*) has been omitted for brevity.

In our baseline scenario (Fig 2, parameters as in Table 1), the model shows a typical pest outbreak originating in the GTA infesting all campgrounds in Ontario, Manitoba and Quebec over 10-20 years. This agrees with the observed timescale of the recent infestation of emerald ash borer (EAB) which entered Ontario in 2002 and now has infested most major populated places in the province [45].

First we discuss the timeseries plot of the baseline parameters (Table 1), where the model variables are averaged over all of the patches for easier visualization (Fig 2). Accordingly, we define $I\left(t\right)=\frac{1}{N}\sum_{i=1}^{N}I_{i}\left(t\right)$, $B\left(t\right)=\frac{1}{N}\sum_{i=1}^{N}B_{i}\left(t\right)$, $L\left(t\right)=\frac{1}{N}\sum_{i=1}^{N}L_{i}\left(t\right)$, to be the average infested tree population at *t*, the average quantity of infested logs at *t*, and the average fraction of local strategists at *t*, respectively. In Fig 2, we find that increasing *U* (the social cost to transport firewood) increases the number of local strategists *L*(*t*) (Fig 2h)—people who choose not to transport firewood between patches— and also reduces the size of the invasion, (Fig 2f) and the average number of infested logs, *B*(*t*) (Fig 2g). Although the reduction in B(t) is significant (as shown by the large differences in light red and dark red time series in Fig 2g), the flattening of the curve for infested trees (Fig 2f) is comparatively less significant. We can compare this with the result of increasing the fraction of infested logs intercepted between patches, *C*_*e*_ (2a,b,c,d). Increasing *C*_*e*_ decreases the number of infested trees, the delays the peak of the outbreak (Fig 2b and 2c). The delay in the peak of the outbreak also appears to cause the lag in *L*(*t*) (Fig 2d). Social incentives appear to be very effective at reducing *B*(*t*) while being less effective at reducing *I*(*t*). This indicates that a shift from transport strategists to local strategists primarily occurs in areas that have already been infested. This effect does not occur with direct interception of infested firewood. Notably, direct interception is difficult to implement effectively, as even after intercepting high proportions of the infested wood transport, the corresponding decrease in *I*(*t*) remains low (Fig 2b).

**Fig 3:**
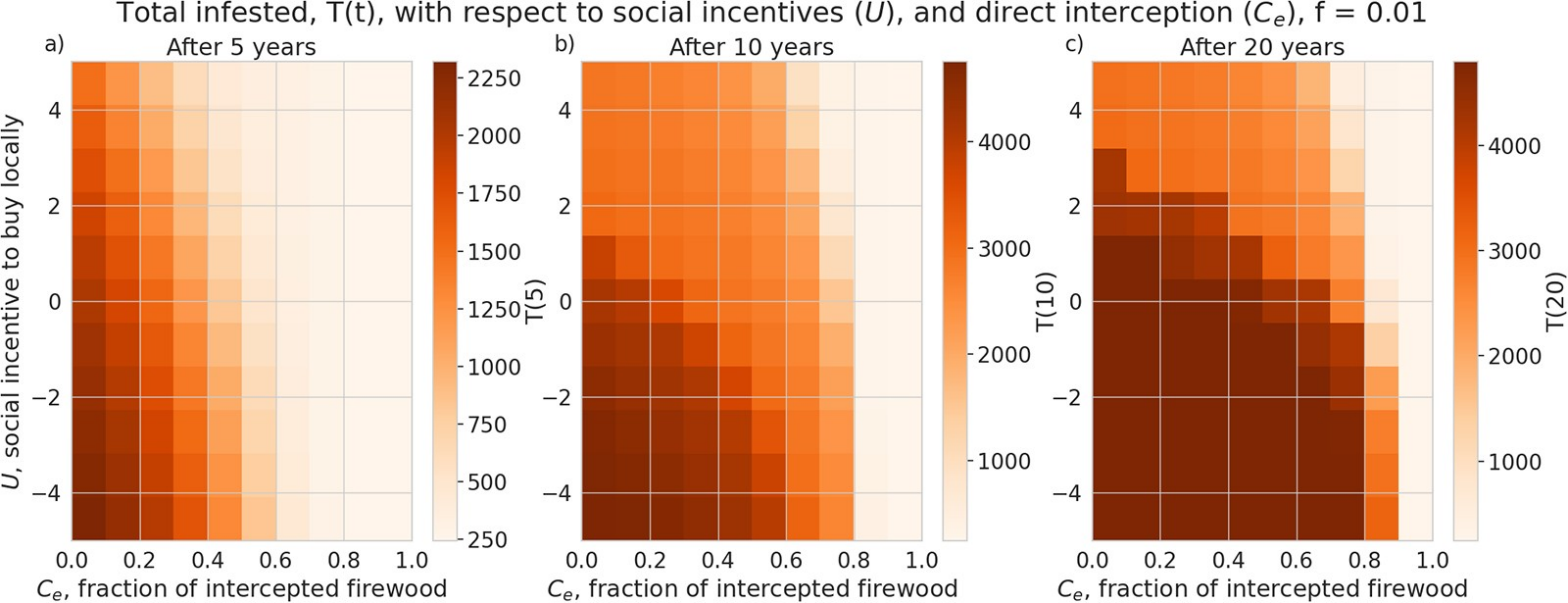
Total infestation per node over 5, 10 and 20 years. Neither increasing *U* nor *C*_*e*_ are effective at long time scales.

In Fig 3 we show the total number of infested trees at time *t*, *T*(*t*), with respect to combinations of *U*, the social cost to transport firewood, and the fraction of infested firewood intercepted, *C*_*e*_. If the fraction of intercepted infested firewood, *C*_*e*_, is greater than 80%, we see a sharp reduction in the total infestation, *T*, even after 20 years (Fig 3c), but lower interception rates have little effect unless the social cost to transport *U* is above the threshold seen in panel c) (Fig 3). Over a shorter time scale, increasing *C*_*e*_ appears to be effective at all interception rates.

**Fig 4:**
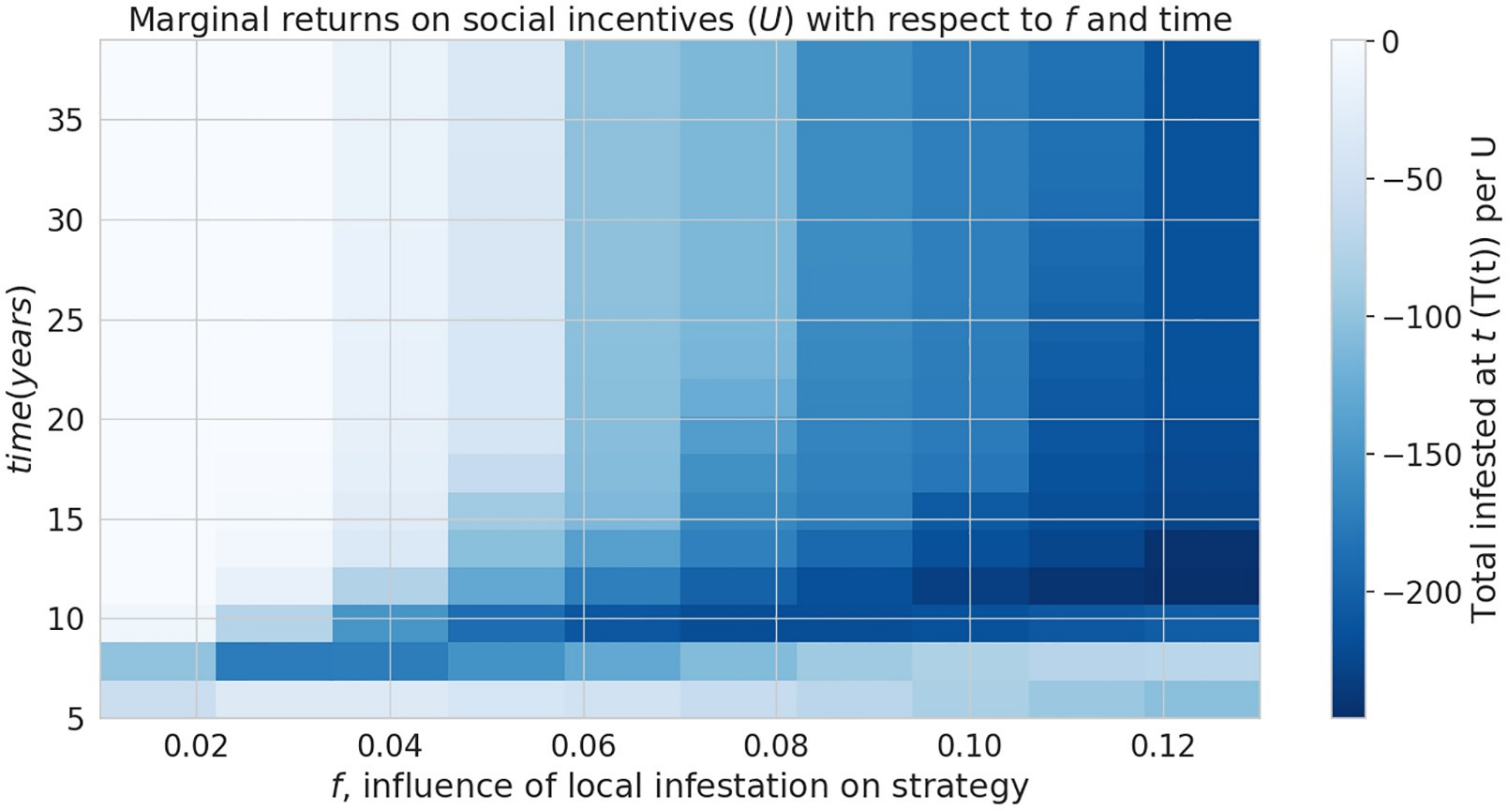
Efficacy of social incentives on infestation after time *T*. Inset graph shows an example of cross-section along the line *f* = 0.11 The influence of infestation on transport strategy, *f*, can hinder the intervention by public outreach, in the long-term (after approximately 20 years). The inset figure illustrates how one column in the heat map, shown by the dotted line, is constructed from the slopes of linear approximations of *T*(*t*) over *U* ∈ [−5, 5]. The blueness of the lines going left to right is a function of their slope, corresponding to the color of the cells in the heatmap.

The parameter *f* controls how the proportion of strategists in a given patch *i* (*L*_*i*_(*t*)) responds to the population of infested trees (*I*_*i*_) in that patch (Eq 8). Since social incentives (such as an intervention to human-mediated pest transport) tend to be less effective because they prevent firewood transport mostly in the areas that have already been colonized by pests (as suggested in Fig 2), we consider how the parameter *f* affects the marginal returns on *U* over time (Fig 4). The shade of the blue region in Fig 4 represents the degree to which increasing *U* is beneficial, corresponding to a negative slope in the linear approximation of the change in *T* with respect to *U* (Fig 4 inset). Similarly, a red cell indicates non-negative slope and therefore a neutral or detrimental marginal effect. We begin to see the benefit of increasing *U* after about 10 years, shown by the transition from lighter blue to dark blue as we move from the bottom of the image to the top (Fig 4. This relationship is only affected slightly by altering the impact of local infestation on local strategy, *f*, where we begin to see slightly detrimental marginal returns after 10 years if *f* < 0.04.

**Fig 5:**
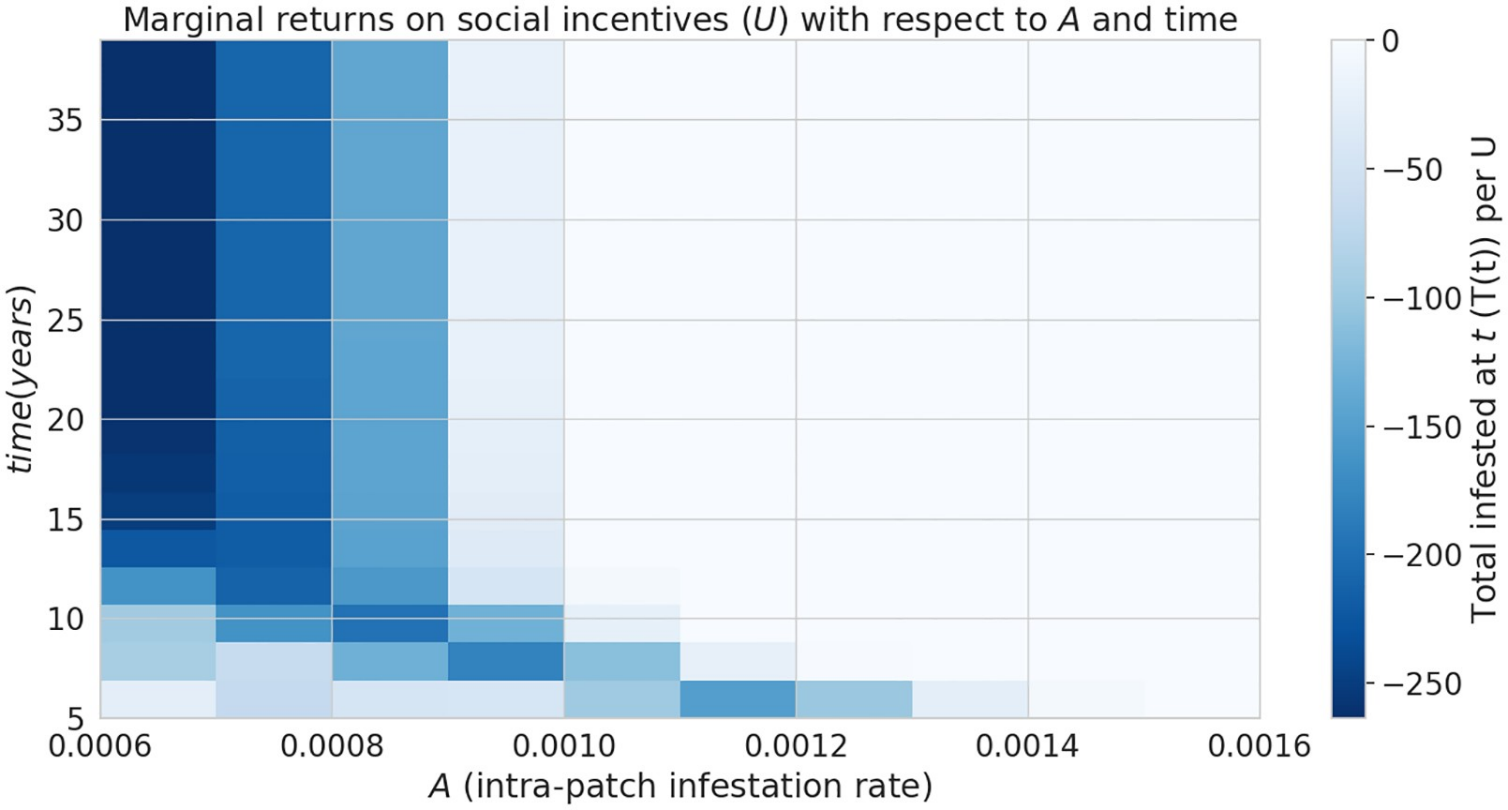
Efficacy of social incentives on infestation after time period *T* with respect to *A*, the intra-patch infestation parameter. This intervention becomes ineffective over time if *A* is sufficiently large.

**Fig 6:**
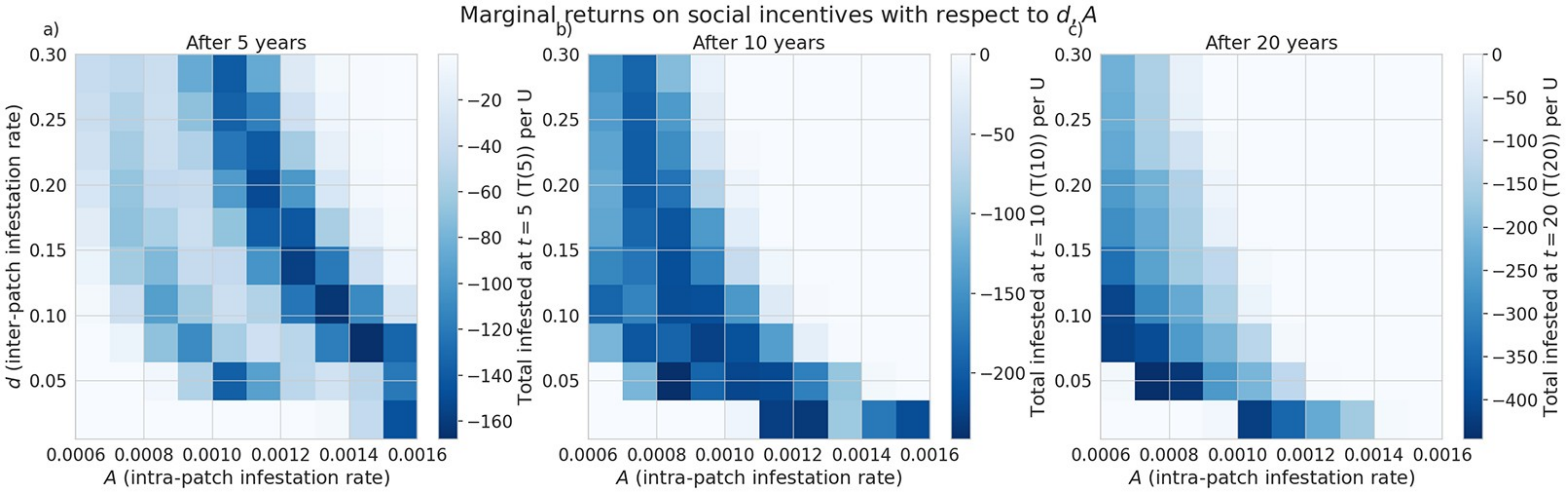
Efficacy of social incentives on infestation after time *T* intra-patch spreading rate *A*, affects infestation outcomes. The social incentive to not transport firewood, *U*, is more effective with lower pest spread rates.

Similarly, we have compared the marginal returns on increasing *U* with respect to the intra-patch transmission rate *A* and time *t* (Fig 5). When *A* is small (*A* ≤ 0.0009, beneficial marginal returns on *U* can be observed over the whole duration of the infestation. We further explore the impact of varying the rate of transmission of infested firewood between patches, *d* (Fig 6). We find a roughly parabola-shaped region in the parameter plane of intra-patch and inter-patch transmission rates (*A* and *d* respectively), above which the marginal returns of increasing *U* are zero or possibly detrimental to the size of the total infested population after 10-20 years. Larger intra-patch transmission rates enable the pest population to establish earlier in a given patch by propagules. We see good marginal return in parameter regimes where few transport strategists (high *L*(*t*)) would reduce the reproductive ratio of the infection below 1. For instance, at the point (*A*, *d*) = (0.00126, 0.103), increasing *U* is able to delay and eventually prevent a second wave, which decreases the total number of infected trees significantly (S1 Fig). If the transmission rates *A*, *d* are high enough that even with no transport strategists, we get a second wave of infection, the effect of increasing *U* can be slightly detrimental (S2 Fig). Panel f) of the aforementioned figures plots the number of patches where *I* ≥ 1 over time, showing that the detrimental effect is largely due to the infection persisting longer in the network.

**Fig 7:**
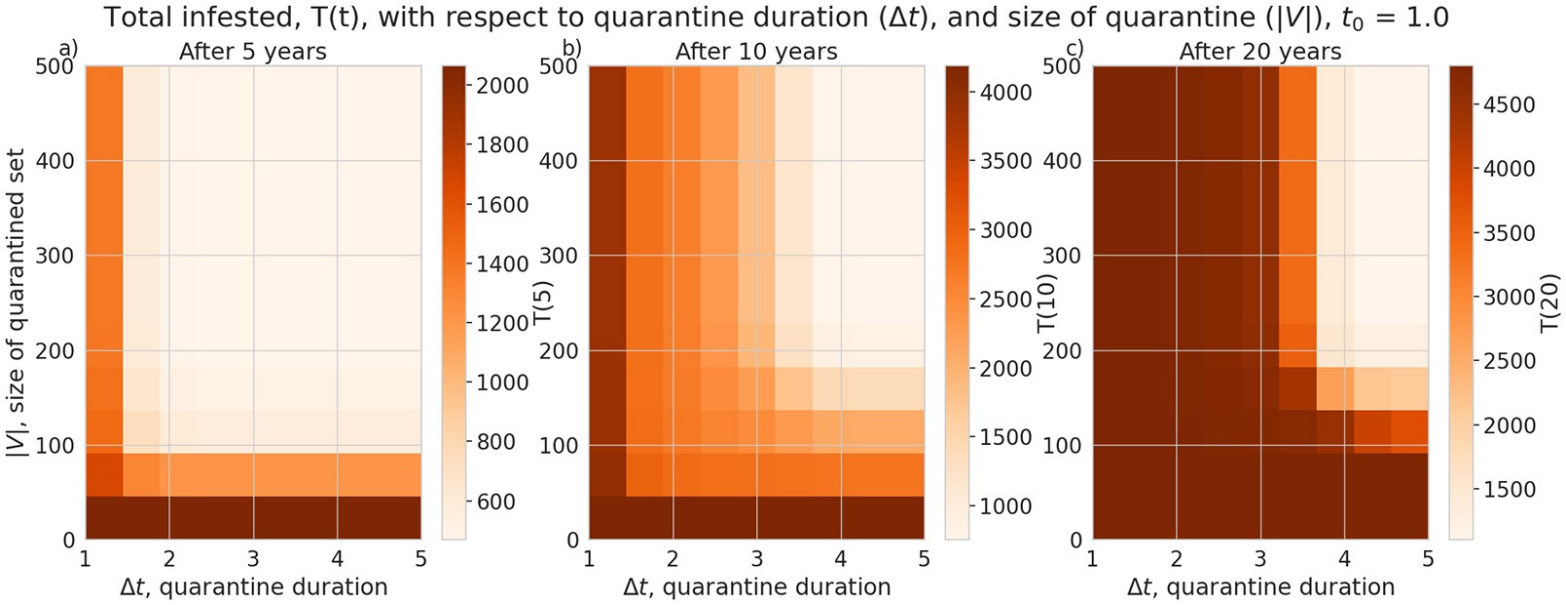
Average total infested trees (*T*(*t*)) after 5, 10 and 15 years (panels a),b), and c) respectively), assuming the quarantine begins one year after the pest is introduced. Total infestation plotted with respect to the number of nodes quarantined (|*V*|) and the length of the quarantine (Δ*t*). The quarantine is effective over 5 years with only 50 patches, provided they are closed for over a year.

**Fig 8:**
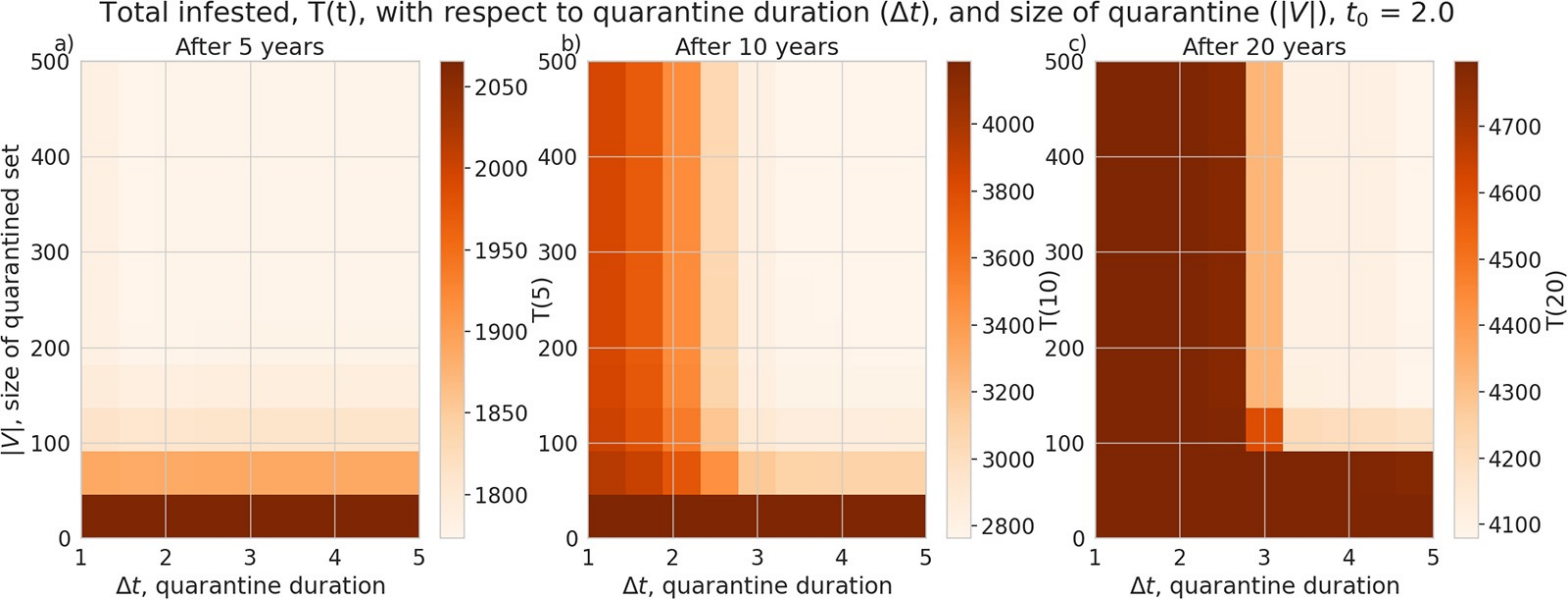
Average total infested trees (*T*(*t*)) after 5, 10 and 15 years (panels a),b), and c) respectively), assuming the quarantine begins two years after the pest is introduced. Total infestation plotted with respect to the number of nodes quarantined (|*V*|) and the length of the quarantine (Δ*t*).

We also explored the effectiveness of patch quarantine by replacing model Eqs (6) and (7) with Eqs (9)–(12). This replacement prevents individual patches (nodes in a set *V*) with the highest betweenness centrality (with respect to the weights *P*_*ij*_) from interacting with their neighbours during the time of the quarantine (*t* ∈ [*t*_0_, *t*_0_ + Δ*t*]). Imposing quarantine on these nodes is expected to have the greatest impact on pest transmission rate. If the quarantine is initiated one year after the pest is introduced into the system (that is, *t*_0_ = 1.0) then we find a significant reduction in total infestation even if only 50 patches are quarantined (|*V*| = 50) assuming they are quarantined for more than a year, shown in Fig 7. However, in our model, we find that quarantines need to be longer than approximately three years, and involve more than 150 nodes to still be effective in reducing the total infested population after 20 years *T*(20). An interesting result in our quarantine plots is that we see a slightly larger range of effective parameter values if the quarantine begins after two years, *t*_0_ = 2.0 (Fig 8), rather than one, *t*_0_ = 1.0. This effect is probably due to the delay in infestation after the model is initialized, which can be seen by the local minimum in the infestation timeseries (Fig 2b and 2f).

## Conclusion

We presented a model coupling human social behaviour regarding transport of infested firewood through recreational travel with a model of the spread of an invasive forest pest. Our main focus was to compare, in relative terms, common measures for slowing the spread of invasive species with firewood transport, such as public outreach campaigns aimed to raise awareness about the problem, and enforcement measures, including inspections at checkpoints to control the movement of firewood, and location-specific quarantine. The model is parameterized with campground reservation data for provincial parks and campgrounds in the provinces of Ontario, Manitoba and Quebec, Canada and incorporated spatial information on the topology and geographical configuration of the camper travel network.

Under the assumptions of our model and a particular camper travel network configuration used in our model, checkpoints to control the movement of untreated firewood are unlikely to be effective at slowing the spread of invasive forest pests with firewood transport given typical moderate levels of funding and long delays in the response measures. We find the rate of interception to halve the total infested tree population after 5 years is about 30% (Fig 3), which is unlikely to be achieved in practice given typical limited budgets and personnel constraints in present-day firewood control programs. Given that our model uses somewhat simplified assumptions and does not account for fine-scale logistical constraints (which are inspectors may face in various spatial locations) the actual rate of interception is likely to be lower in practical conditions. While a previous study [18] that used a similar model has demonstrated that social incentives may improve outcomes in a two-patch model under equilibrium conditions, we have found that in our complex landscape network, the outcomes of infestation and invasion control measures are highly dependent on the time scale and the characteristics of the invaders, such as the inter-patch and intra-patch infestation rate. Social incentives (which aim to decrease the transport of firewood, *U*), are generally able to reduce the infestation rate in the short term but its effectiveness is highly dependent on the ability of the pest to spread and infest other locations (Figs 5 and 6) under the conditions we have explored. Humans in our model tend to reduce their transport of firewood between patches in already infested areas, which causes the pest to persist longer in the network (Fig 2). Our results show that there could exist a threshold in the pest transmission rate *A* and the proportion of the infested wood which is turned into firewood, *d* (Fig 6). Below this threshold, it would not be beneficial to increase social outreach (i.e., increase *U*). This insight could be helpful in determining the spatial allocation of firewood movement control efforts for a particular pest species. We have also found that the location-specific quarantines that aim to restrict the movement of firewood to/from a particular location, might only be effective at slowing the invasion spread if a sufficiently large number (at least 140 in our case) of highly connected locations is quarantined, and the quarantine is established at early stages of infestation (Figs 7 and 8).

Given the typical cost limitations and logistics constraints faced by today’s firewood control programs, and the assumptions made in our modeling framework, it is unlikely that local quarantine measures could significantly slow the spread of invasive pests through firewood unless drastic control and quarantine measures are undertaken. Public outreach campaigns, while helping increasing awareness of problem, cannot reliably slow the spread of pests within the parameter values tested, when the invasion spreads through a network based on camper travel data in Manitoba, Ontario and Quebec. Within our model, public outreach could be more effective for slow-spreading pests when the organism is able to kill host trees quickly but does not have significant spread capacity (that is, the inter-patch and intra-patch infestation rates are sufficiently small). Direct intervention, such as checkpoint inspections for illegally transported firewood, is also not an option, because meaningful outcomes can only be achieved if significant fractions of firewood transports can be intercepted. We find that patch quarantine is effective at slowing, or even stopping, the spread of an invasive forest pest when a large number of highly-connected patches are quarantined, for a long enough period. Our results in general terms agree with a present-day situation when numerous outreach and local quarantine measures had limited impact on illegal transport of firewood by campers and failed to slow the spread of wood-boring pests transported with untreated firewood. Our results also indicate that the enforcement campaigns aimed to intercept illegal movement of untreated firewood can only be effective if implemented at very large spatial scales in timely fashion (which, in turn, would require massive amounts of funding and personnel support).

There are some shortcomings to our model that could be addressed in future work. The interventions we study do not have spatial or time specifications for individual locations in the camper travel network. Deciding where and when, to deploy the outreach and enforcement measures in a particular location would be a major enhancement of the model. Second, our model depicted a general problem of an invasive pest spreading with untreated firewood moved by recreational travelers. To adapt the problem to a particular pest species, a more specialized spread model will be required. We simplified the model by assuming that each infested patch provides similar propagule pressure to recreational travellers leaving the infested site. This assumption was made because no data about the actual proportions of infested wood carried by recreational travellers leaving the infested sites were available. Also, our analysis did not offer much insight at the level of individual spatial locations in a camper travel network. A simpler mechanistic model that applies unique pest control decisions at individual spatial locations could potentially address that aspect. Another possible way to simplify the model would be to remove the tree growth dynamics —since it operates on a longer time scale than the infestation spread— and so an invasion model without the forest growth component could be a reasonable approximation for short-term planning horizons. This will be the focus of future efforts.

## Supporting information

S1 Fig Time evolution of model variables for various values of *U* where (*A*, *d*) = (0.0009, 0.038). (PDF)

S2 Fig Time evolution of model variables for various values of *U* where (*A*, *d*) = (0.00126, 0.103). (PDF)

## References

[1] Wilson JR, Dormontt EE, Prentis PJ, Lowe AJ, Richardson DM. Something in the way you move: dispersal pathways affect invasion success. Trends in ecology & evolution. 2009;24(3):136–144. 10.1016/j.tree.2008.10.007 19178981

[2] Jacobi W, Goodrich B, Cleaver C, et al Firewood transport by national and state park campers: a risk for native or exotic tree pest movement. Arboriculture and Urban Forestry. 2011;37(3):126.

[3] Tobin PC. Cost analysis and biological ramifications for implementing the gypsy moth Slow the Spread Program. Gen Tech Rep NRS-37 Newtown Square, PA: US Department of Agriculture, Forest Service, Northern Research Station 21 p. 2008;37.

[4] National Invasive Species Council (NISC). Fiscal Year 2007 Interagency Invasive Species Performance Budget; 2007. Online source. Available from: https://edit.doi.gov/sites/doi.gov/files/migrated/invasivespecies/org_collab_budget/upload/NISC-FY2007-Crosscut-Budget-Summary.pdf.

[5] Kolar CS, Lodge DM. Progress in invasion biology: predicting invaders. Trends in ecology & evolution. 2001;16(4):199–204. 10.1016/S0169-5347(01)02101-2 11245943

[6] Kovacs KF, Haight RG, McCullough DG, Mercader RJ, Siegert NW, Liebhold AM. Cost of potential emerald ash borer damage in US communities, 2009–2019. Ecological Economics. 2010;69(3):569–578. 10.1016/j.ecolecon.2009.09.004

[7] Kovacs KF, Haight RG, Mercader RJ, McCullough DG. A bioeconomic analysis of an emerald ash borer invasion of an urban forest with multiple jurisdictions. Resource and Energy Economics. 2014;36(1):270–289. 10.1016/j.reseneeco.2013.04.008

[8] Poland TM, McCullough DG. Emerald ash borer: invasion of the urban forest and the threat to North America’s ash resource. Journal of Forestry. 2006;104(3):118–124.

[9] Buck JH, Marshall JM. Hitchhiking as a secondary dispersal pathway for adult emerald ash borer, Agrilus planipennis. Gt Lakes Entomol. 2009;41:197–199.

[10] Koch FH, Yemshanov D, Haack RA, Magarey RD. Using a network model to assess risk of forest pest spread via recreational travel. PloS one. 2014;9(7):e102105 10.1371/journal.pone.0102105 25007186 PMC4090238

[11] Liebhold AM, Tobin PC. Population ecology of insect invasions and their management. Annu Rev Entomol. 2008;53:387–408. 10.1146/annurev.ento.52.110405.091401 17877456

[12] Prasad AM, Iverson LR, Peters MP, Bossenbroek JM, Matthews SN, Sydnor TD, et al Modeling the invasive emerald ash borer risk of spread using a spatially explicit cellular model. Landscape ecology. 2010;25(3):353–369. 10.1007/s10980-009-9434-9

[13] Cordell HK, Betz C, Green GT. Nature-based outdoor recreation trends and wilderness. International Journal of Wilderness, August 2008, Volume 14, Number 2, Page 7-13. 2008;.

[14] Cordell HK. The latest trends in nature-based outdoor recreation. Forest History Today, Spring 2008. 2008.

[15] Cordell HK. Outdoor recreation trends and futures: a technical document supporting the Forest Service 2010 RPA Assessment. Gen Tech Rep SRS-150 Asheville, NC: US Department of Agriculture Forest Service, Southern Research Station, 167 p. 2012;150:1–167.

[16] Eagles PF. Trends in park tourism: Economics, finance and management. Journal of sustainable tourism. 2002;10(2):132–153. 10.1080/09669580208667158

[17] Siikamäki J. Contributions of the US state park system to nature recreation. Proceedings of the National Academy of Sciences. 2011;108(34):14031–14036. 10.1073/pnas.1108688108 PMC3161538 21831838

[18] Barlow LA, Cecile J, Bauch CT, Anand M. Modelling interactions between forest pest invasions and human decisions regarding firewood transport restrictions. PLoS One. 2014;9(4):e90511 10.1371/journal.pone.0090511 24736497 PMC3987997

[19] Tobin PC, Diss-Torrance A, Blackburn LM, Brown BD. What does “local” firewood buy you? Managing the risk of invasive species introduction. Journal of economic entomology. 2010;103(5):1569–1576. 10.1603/EC10140 21061955

[20] Peterson K, Diss-Torrance A. Motivation for compliance with environmental regulations related to forest health. Journal of environmental management. 2012;112:104–119. 10.1016/j.jenvman.2012.06.023 22890000

[21] Haack RA, Law KR, Mastro VC, Ossenburgen HS, Raimo BJ. New York’s battle with the Asian long-horned beetle. Journal of Forestry 95 (12): 11–15. 1997;2(12).

[22] Petrice TR, Haack RA. Effects of cutting date, outdoor storage conditions, and splitting on survival of Agrilus planipennis (Coleoptera: Buprestidae) in firewood logs. Journal of Economic Entomology. 2006;99(3):790–796. 16813313 10.1603/0022-0493-99.3.790

[23] United States Department of Agriculture Animal and Plant Health Inspection Service. Risk Assessment of the Movement of Firewood within the United States; 2011. Available from: https://www.aphis.usda.gov/import_export/plants/plant_imports/firewood/firewood_pathway_assessment.pdf.

[24] Haack RA, Petrice TR, Wiedenhoeft AC. Incidence of bark-and wood-boring insects in firewood: a survey at Michigan’s Mackinac Bridge. Journal of Economic Entomology. 2010;103(5):1682–1692. 10.1603/EC10041 21061968

[25] Ontario Ministry of Natural Resources. Are you moving firewood? Important information you should read; 2011. Available from: http://www.mnr.gov.on.ca/en/Business/Forests/2ColumnSubPage/STEL02_167014.html.

[26] Tegner M, Dayton P. Population structure, recruitment and mortality of two sea urchins (Strongylocentrotus franciscanus and S. purpuratus) in a kelp forest. Marine Ecology Progress Series. 1981; p. 255–268. 10.3354/meps005255

[27] Ludwig D, Jones DD, Holling CS, et al Qualitative analysis of insect outbreak systems: the spruce budworm and forest. Journal of animal ecology. 1978;47(1):315–332. 10.2307/3939

[28] Ali Q, Bauch CT, Anand M. Coupled Human-Environment Dynamics of Forest Pest Spread and Control in a Multi-Patch, Stochastic Setting. PloS one. 2015;10(10):e0139353 10.1371/journal.pone.0139353 26430902 PMC4592071

[29] Reid N. Roadside checkpoints to monitor illegal firewood transport in New Hampshire. Concord Monitor. 2017;.

[30] Weber L. Checkpoint tries to halt wood traffic. The Toledo Blade. 2006;.

[31] Nearing B. Checkpoint tries to halt wood traffic. timesunion. 2012;.

[32] Henderson KA, Anand M, Bauch CT. Carrot or stick? Modelling how landowner behavioural responses can cause incentive-based forest governance to backfire. PloS one. 2013;8(10):e77735 10.1371/journal.pone.0077735 24204942 PMC3813756

[33] Bauch CT. Imitation dynamics predict vaccinating behaviour. Proceedings of the Royal Society B: Biological Sciences. 2005;272(1573):1669–1675. 10.1098/rspb.2005.3153 PMC1560180 16087421

[34] Hofbauer J, Sigmund K. Evolutionary games and population dynamics. Cambridge university press; 1998.

[35] Muirhead JR, Leung B, van Overdijk C, Kelly DW, Nandakumar K, Marchant KR, et al Modelling local and long-distance dispersal of invasive emerald ash borer Agrilus planipennis (Coleoptera) in North America. Diversity and Distributions. 2006;12(1):71–79. 10.1111/j.1366-9516.2006.00218.x

[36] Gottwald TR, Hughes G, Graham JH, Sun X, Riley T. The citrus canker epidemic in Florida: the scientific basis of regulatory eradication policy for an invasive species. Phytopathology. 2001;91(1):30–34. 10.1094/PHYTO.2001.91.1.30 18944275

[37] Yemshanov D, Haight RG, Koch FH, Lu B, Venette R, Lyons DB, et al Optimal allocation of invasive species surveillance with the maximum expected coverage concept. Diversity and Distributions. 2015;21(11):1349–1359. 10.1111/ddi.12358

[38] Satake A, Leslie HM, Iwasa Y, Levin SA. Coupled ecological–social dynamics in a forested landscape: Spatial interactions and information flow. Journal of theoretical biology. 2007;246(4):695–707. 10.1016/j.jtbi.2007.01.014 17376488

[39] Freeman LC. A set of measures of centrality based on betweenness. Sociometry. 1977; p. 35–41. 10.2307/3033543

[40] Brandes U. On variants of shortest-path betweenness centrality and their generic computation. Social Networks. 2008;30(2):136–145. 10.1016/j.socnet.2007.11.001

[41] Wang Z, Bauch CT, Bhattacharyya S, d’Onofrio A, Manfredi P, Perc M, et al Statistical physics of vaccination. Physics Reports. 2016;664:1–113.

[42] Hagberg A, Swart P, S Chult D. Exploring network structure, dynamics, and function using NetworkX. Los Alamos National Lab.(LANL), Los Alamos, NM (United States); 2008.

[43] Rackauckas C, Nie Q. Differentialequations. jl–a performant and feature-rich ecosystem for solving differential equations in julia. Journal of Open Research Software. 2017;5(1).

[44] Turgeon JJ, Orr M, Grant C, Wu Y, Gasman B. Decade-Old Satellite Infestation of Anoplophora glabripennis Motschulsky (Coleoptera: Cerambycidae) Found in Ontario, Canada Outside Regulated Area of Founder Population. The Coleopterists Bulletin. 2015;69(4):674–679. 10.1649/0010-065X-69.4.674

[45] Canadian Food Inspection Agency (CFIA). https://www.inspection.gc.ca/plants/plant-pests-invasive-species/insects/emerald-ash-borer/areas-regulated/eng/1347625322705/1367860339942; 2019.

