# Correction: Go big or go home: A model-based assessment of general strategies to slow the spread of forest pests via infested firewood

**DOI:** 10.1371/journal.pone.0261425

**Published:** 2021-12-09

**Authors:** Peter C. Jentsch, Chris T. Bauch, Denys Yemshanov, Madhur Anand

In Fig 4 the inset plot is missing. Please see the correct Fig 4 here.

**Fig 4:**
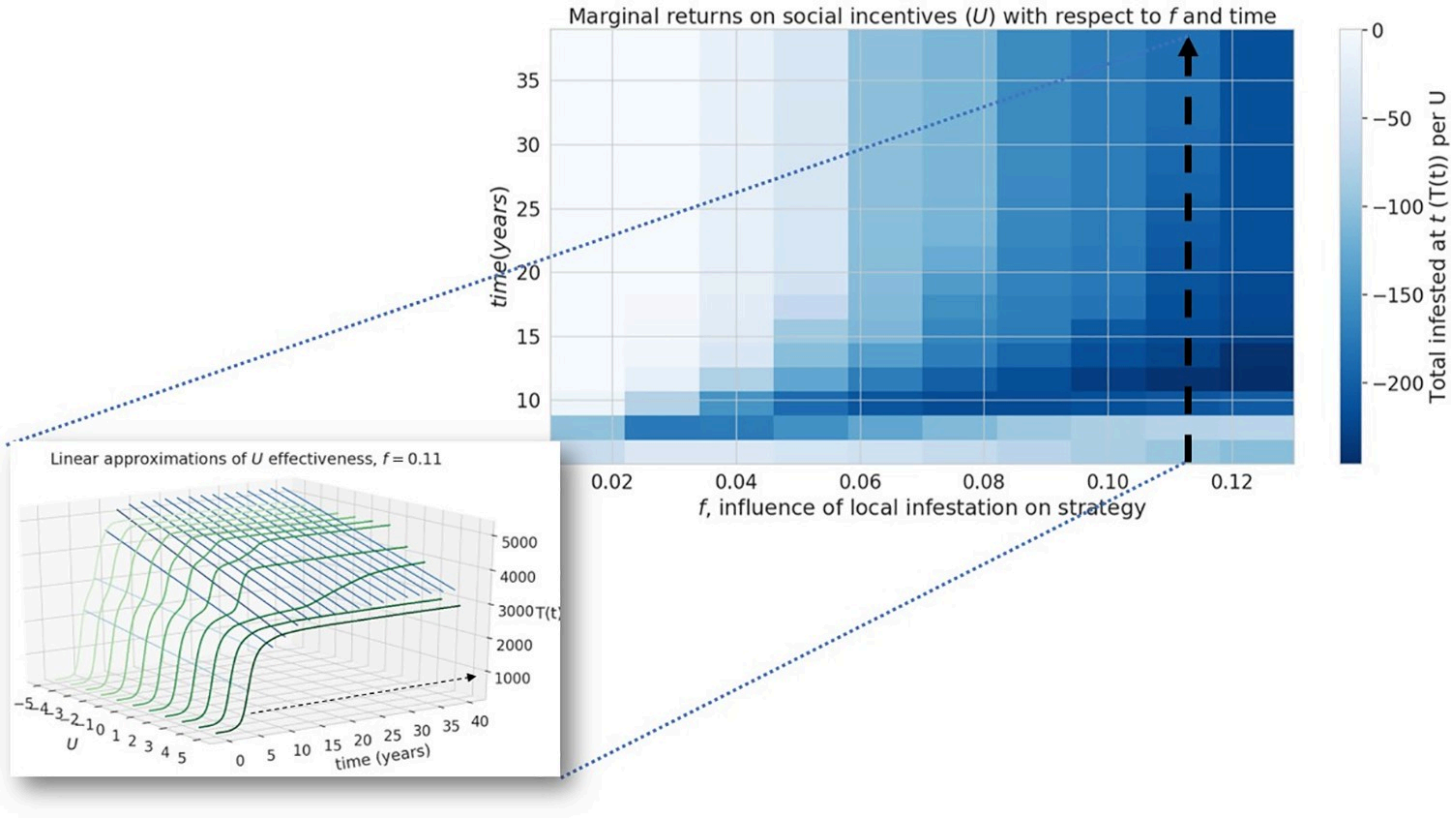
Efficacy of social incentives on infestation after time *T*. Inset graph shows an example of cross-section along the line *f* = 0.11 The influence of infestation on transport strategy, *f*, can hinder the intervention by public outreach, in the long-term (after approximately 20 years). The inset figure illustrates how one column in the heat map, shown by the dotted line, is constructed from the slopes of linear approximations of *T*(*t*) over *U* ∈ [−5, 5]. The blueness of the lines going left to right is a function of their slope, corresponding to the color of the cells in the heatmap.
