# Supplementary figures and images for: Go big or go home: A model-based assessment of general strategies to slow the spread of forest pests via infested firewood
Source: PLoS One. 2020 Sep 15;15(9):e0238979. doi: 10.1371/journal.pone.0238979 (PMC7491730; doi:10.1371/journal.pone.0238979)

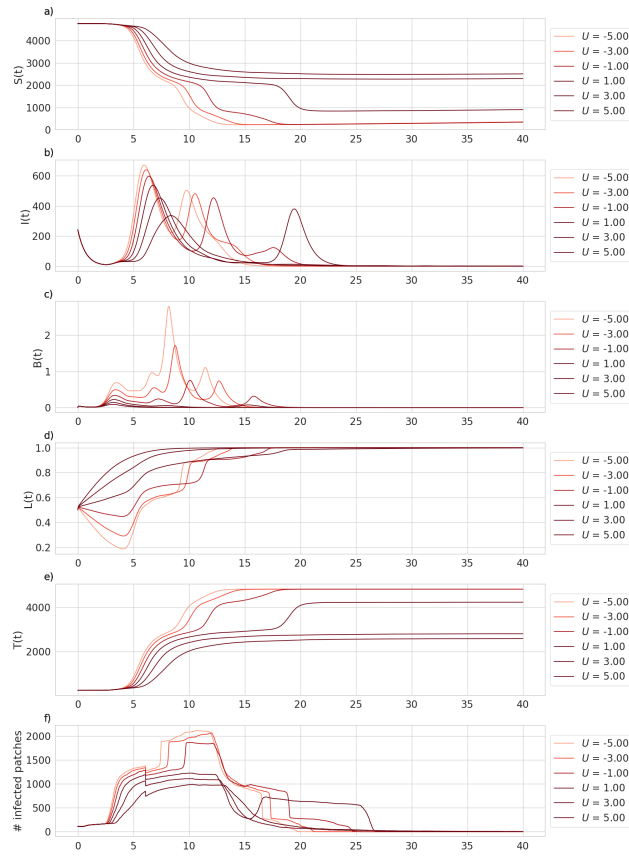

S1 Fig: Time evolution of model variables for various values of  $U$  where  $(A, d) = (0.0009, 0.038)$ .

Supplement: S1 Fig — (PDF) [file pone.0238979.s001.pdf]

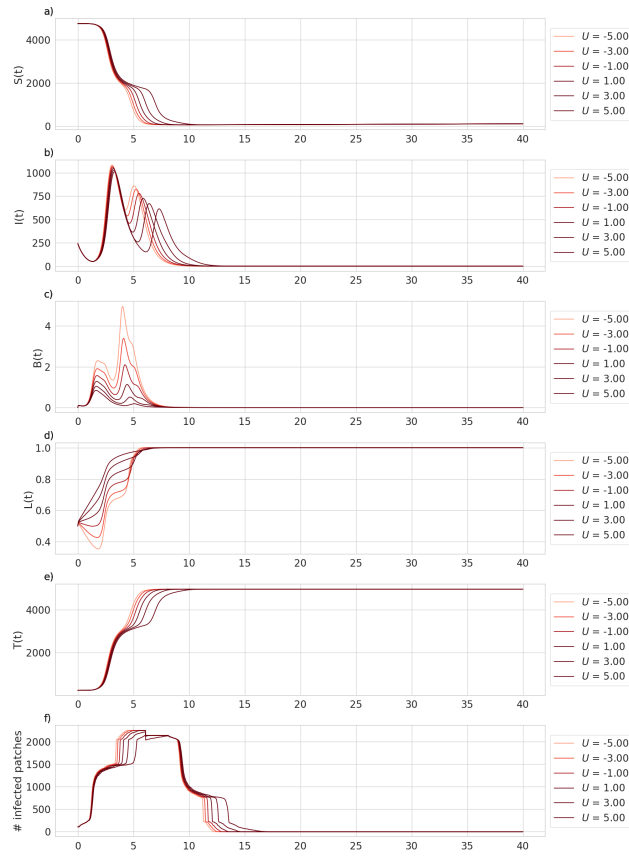

S2 Fig: Time evolution of model variables for various values of  $U$  where  $(A, d) = (0.00126, 0.103)$ .

Supplement: S2 Fig — (PDF) [file pone.0238979.s002.pdf]
