# Supplementary material for: Go big or go home: A model-based assessment of general strategies to slow the spread of forest pests via infested firewood
Source: PLoS One. 2020 Sep 15;15(9):e0238979. doi: 10.1371/journal.pone.0238979 (PMC7491730; doi:10.1371/journal.pone.0238979)
Supplement: Attachment — Submitted filename: Jentsch_2020_pone_review.pdf [file pone.0238979.s003.pdf]

Review of Jentsch et al. (2020)

This paper extends the Barlow et al. (2014) model of forest pest invasion by incorporating firewood transport across a large network of recreational sites. In the present model, the rates at which firewood is transported between sites depend on quarantine efforts, firewood interception efforts, and social costs.

In order to manage the complexity of the model, simple models were used to describe the forest and infection dynamics. The proposed model seems like it might be reasonable. However, several errors were made in the model description, which make it difficult to follow its development, and the descriptions of variables and parameters are often imprecise and inconsistent. This makes it difficult (and frustrating) to evaluate the model and the parameter value choices. It really needs to be fixed. Some of the problems in the model development include:

1. In line 111, P_{ij} is introduced and described as quantifying movements between origin locations I and recreational destinations j. Elsewhere it is used to indicate movements from j to i.
2. In line 111, P_{ij} is defined as a relative frequency of camper movements, in table 1 it is described as the average number of trips from patch j to patch i. These are not the same, and neither definition is very precise. Please make the definition precise. It would help to tell the reader what the units are, for example.
3. If P_{ij} is defined as in table 1, and d defined as in table 1, then it does not seem that the units for the last term in equation (9) are correct (or in other similar terms in other equations). I suspect that this can be solved by giving a more precise definition of these terms.
4. S_i and I_i are not defined in the text near equation (1). Precise definitions here are VERY important. Are these numbers, densities? Does I include fallen infected wood? If so, should it affect intraspecific competition in the way it does in the model? If it doesn't, shouldn't there be another variable describing local fallen wood, which can infect susceptible trees? If I and S are numbers, are there the same numbers of trees at each node? If not, K should vary from site to site as in the Barlow et al model. If they are densities, then K could reasonably be constant across sites, but then one density unit of wood transported from site 1 would represent a different density at site 2. The definitions of these and other variables in the model affect the meanings and values of the parameters, imply particular model assumptions, and even determine whether the model structure is correct. Have these things been carefully considered? There is no evidence of this in the text. Inconsistencies in the definitions that are provided do not increase my confidence.
5. Equation (2) should probably read kx instead of k(x), since k is a constant
6. It should be stated that k is a constant near equation (2)
7. I_a does not determine the smallest viable population. At I_a, the phi function has the value ½. In the Barlow paper, a step function was used for phi, so the parameter has a

different interpretation. Also, it does not really refer to a pest population, but rather a population of infested trees. This should be clarified.

8. P_{ij} is defined again, and imprecisely at that, in line 147. Please define it once and be precise.
9. The description immediately before and after equations (1-2) seem to suggest that the infestation term describes transmission from firewood. The model suggests that this term represents infestation resulting from interaction between susceptible trees in patch I and infested trees in patch i. Are infested trees being equated with local firewood? Please be precise. If so, does it make sense for them to be affecting intraspecific competition in the same way that susceptible trees do in the logistic growth term?
10. Why is C_e being defined again in line 148? It was defined on line 118.
11. The parameter d is introduced in the equation after line 150, but it is not defined
12. L_i is defined as a number of visitors in line 146, then used as a proportion in the equation after line 150.
13. What are the units for the expression following line 150?
14. U is defined differently in lines, 156, 172, and 126. Please be precise, consistent, and define variables once.
15. The parameters n and f are not defined in the equations after line 160
16. The second equation after line 160 is inconsistent with Barlow et al. (2014) and does not make sense as written here. In particular the $-fI_i$ belongs in the first equation but not the second one
17. Line 161 promises an explanation that never seems to come.
18. Biologically, the equation after line 166 should have P_l-P_t instead of its opposite. As written, it is also inconsistent with the Barlow et al. paper.
19. In line 167, it should be specified that C_e=0 in (4-6)
20. The arguments of phi should be I_i-I_a in equations 4-5 to be consistent with the notation in the present work.
21. The definition of B_i in line 176 is not consistent with how it is used in the model. It seems that B_i must be the amount of imported firewood, not the amount of firewood, since there is no local source term. This also seems to suggest that I_i is being treated as local firewood rather than as infested trees.
22. Gamma is used in equations (3) and (7). The text even points out that these things are assumed to be the same. So why are they different (alpha and epsilon) in equations (9-10). If alpha and epsilon are to be used, then they need to be defined.
23. The parameter s that appears in equation (11) needs to be defined in the text.
24. If the definitions of d and P_{ij} in table 1 are to be taken seriously, it seems that there should be another constant multiplying the transport terms. The units don't make sense. As the definitions are tightened up throughout the paper, please reconsider these terms. It is hard to evaluate them when the definitions are not clear.
25. Does gamma (or epsilon and alpha) include loss due to burning?
26. The betweenness centrality does not seem to take the P_{ij}s into account, just the fact that there are edges connecting the nodes. It is worth pointing this out specifically, since it seems like the P_{ij}s should affect the quarantine decision.

27. The definition of g(i) would be more readable if the subscript for the sum in the definition had a semicolon between t and s.
28. In equation (15) there should not be an H_c factor in the first sum.
29. Table 1 does not include a value for K.

Given the lack of precision in the model description, it is impossible to determine the precise meanings of many of the parameters. Thus it is impossible to assess whether reasonable values are being used. The approach to choosing parameter values is described in the 'Parameterization' section. It seems that the approach was to try some values to see what would give reasonable predictions of the dynamics. This does not mean that the parameter values are reasonable. The results include explorations in which values are varied over certain ranges, but are those ranges meaningful? It is difficult to tell when the reader cannot even decipher, for example, the exact meaning of the parameter d. If the values are reasonable, then the results are interesting. However, as the paper stands now, it is difficult to find them compelling or even relevant.

In line 290, what is total infected population? In line 317, the effects of different values of the sum A+d are discussed. A is small relative to d. It is not really the sum that matters here, it is the relative sizes of the individual parameters. Changing A, does not change A+d much, but it does have a large effect on the marginal return of increasing U. Changing d by the same amount (same A+d) has little effect on the marginal return of increasing U.

There are at least two places in the results where the marginal return figures are used as justification for some statement about the dynamics of the system (line 324, 334). The figures do not support these sorts of statements. For example, figure 8 shows the marginal return of adding more quarantine nodes, but it does not show that the existing nodes are ineffective.

Why is there so much focus on the marginal benefit of increasing U or |V| (5 of 8 figures)? This is interesting, but it seems to be of secondary importance when trying to understand the effects of policies on the spread of the invasion. Why not focus more on the effects on directly relevant quantities, such as the size of the invasion or the time required to reach 50% infection, etc.

There are several typos and problems with wording throughout the paper. Here are some that I noticed:

1. Abstract should read 'awareness of the problem' and later 'lack of serious funding'
2. Line 12 'In North America'
3. Line 31 'have been undertaken'
4. Line 32 'educate the general public'
5. Line 60 ..
6. Line 63-64. Delete 'over long distances'
7. Sentence starting on line 64 seems out of place
8. Line 98 [0,N] has N+1 integers

9. Line 99 split sentence '..social dynamics. That is, it..'
10. The sentence ending on line 106 should probably include citations
11. The sentence ending on line 115 is unclear. Would it make sense if 'to a location i' is replaced with 'from that location'
12. Line 117 C_e inequalities should be reversed
13. Line 119 'movement of the infested'
14. Line 133 delete 'an upper bound'. Populations can exceed their carrying capacities.
15. Line 147 'does' should be 'do' and 'buys' should be 'buy' and 'locally.' should end the sentence
16. Line 156 'and C_l'
17. Line 212 'We multiply'
18. Line 216 'not under quarantine'
19. Line 216 'require us to distinguish'
20. Color choices in Fig. 2 make it difficult to distinguish curves from the background.
21. Line 300 section what?
