# Supplementary material for: Go big or go home: A model-based assessment of general strategies to slow the spread of forest pests via infested firewood
Source: PLoS One. 2020 Sep 15;15(9):e0238979. doi: 10.1371/journal.pone.0238979 (PMC7491730; doi:10.1371/journal.pone.0238979)
Supplement: Attachment — Submitted filename: Jentsch_2020_R1_pone_review.pdf [file pone.0238979.s005.pdf]

This revision has been substantially improved over the original submission. I still have some concerns, including major ones about the nature of the conclusions.

1. It is not clear from the definition if it is assumed that
$$\sum_{i=1}^{N} P_{ij} = 1$$
or not. If so, then state it, because this will clarify the definition of P_{ij}. If not, then please be even more precise in your definition for P_{ij}.
2. It seems that the model should take into account the fact that the total amount of firewood exported from a site depends on the number of people leaving the site, not just the number of infected trees there. Unless the sum mentioned above is something other than 1, it does not seem that the model is accounting for this. In this case, this is a limitation of the approach, and it should be addressed in the text.
3. How does \phi model density dependence? What sort of density dependence is it accounting for? It makes sense to me if \phi takes into account that there needs to be a sufficient number of pests attacking susceptible trees to overwhelm tree defenses and successfully infest the tree. If this is the sort of density dependence that \phi is modeling then the argument should be (I_i+B_i-I_a) instead of (I_i-I_a), since infested firewood is also contributing pests (in exactly the same way as infested trees).
4. One thing that seems important in figure 2 is that increasing C_e delays the initial peak in the infection. Increasing U does not have the same effect, probably due to a lag, since increasing U must result in L increasing before importation slows down. The shift in the initial peak in I as C_e increases is probably what leads to the delay in the increase in L (with increasing C_e) in figure 2d. Increasing C_e is probably not decreasing L directly. It is just delaying its increase by delaying the peak in I.
5. Can you explain why increasing U would have a detrimental effect (increasing T) for some parameter values (Figs 4-6)? It seems like there should be some deeper explanation in terms of the dynamics of the infection (what would figure 2e-h look like in these cases?). It seems like at least some attempt should be made to explain this beyond just noting that it happens. This sort of understanding would add value to the paper.
6. The authors did not satisfactorily address my comment regarding the focus on the marginal return on social incentives (basically dT(t)/dU). Focusing on this quanity may be obscuring more relevant dynamics. For example, if dT/dU is positive the infection peak may occur earlier but result in less infection when integrated over time. This could invalidate the statement starting in line 500. Even if dT/dU is positive, there may be more important beneficial effects of increasing U. Addressing my comment 5 may also help to address comment 6.
7. The authors did not satisfactorily address my comment regarding parameter choices and their effect on making specific claims/predictions. They addressed it by stating that their ‘goal was not to provide predictions but to find insight into the behavior of these systems on networks’. However, their conclusions seem to imply some specific management recommendations (or at least I am afraid that they will be interpreted that

way). Their conclusions are valid only if their parameter values are, and they have not justified their parameter choices. Here are some of the specific conclusions (paraphrased):

a. Checkpoints are unlikely to be effective
b. The total rate of interception to halve the infestation is 30%
c. It is unlikely that quarantine measures could be effective
d. At least 25% of firewood transports need to be intercepted to be effective

Minor comments (line numbers refer to version of document with changes tracked):

1. Line 4: replace “on” with “for” or “to”
2. Line 129-131. This sentence is not clear. It does not even seem necessary, so I recommend removing it.
3. Line 148. As defined, the function is not a “shifted” sigmoid. Remove “shifted”.
4. Line 161. Remove “in”
5. Line 169. Replace “importation” with “exportation”
6. Near line 183. The equation for P_t should have (0.5-L_i) instead of (L_i-0.5)
7. Near line 226. You misinterpreted my comment. The semicolon should go in the subscript for \Sigma. That is, it should read $i \neq s \neq t; s,t \in G$
8. Line 228. It should be stated that the weights are the P_{ij} s
9. Lines 226-228. Be specific about how g_{st} and g_{st}(i) depend on the P_{ij}. (Write the equations)
10. Equations 11-12. Replace epsilon with gamma
11. Lines 242-245. This sentence is unnecessary (obvious/redundant)
12. Lines 254-256. This sentence is unnecessary (redundant)
13. Line 378. Remove “is”
14. Line 449 needs a verb.
15. Line 449. “invasion speed” usually refers to spatial spread rate. That is not what figure 7 shows. Maybe just use “total infestation”
16. Line 489. “is” should be “are”
