# Supplementary material for: Go big or go home: A model-based assessment of general strategies to slow the spread of forest pests via infested firewood
Source: PLoS One. 2020 Sep 15;15(9):e0238979. doi: 10.1371/journal.pone.0238979 (PMC7491730; doi:10.1371/journal.pone.0238979)
Supplement: Attachment — Submitted filename: Plos_reviewer_response_2.pdf [file pone.0238979.s006.pdf]

We would like to thank the reviewers and editor for their invaluable comments and suggestions. Please see our replies to comments below. Corresponding changes to the text are in italics.

1. It is not clear from the definition if it is assumed that $\sum_{i=1}^{N} P_{i,j} = 1$ or not.If so, then state it, because this will clarify the definition of $P_{i,j}$. If not, then please be even more precise in your definition for $P_{i,j}$.

   It is the case that $\sum_{i=1}^{N} P_{i,j} = 1$, we have added this to the text.

2. It seems that the model should take into account the fact that the total amount of firewood exported from a site depends on the number of people leaving the site, not just the number of infected trees there. Unless the sum mentioned above is something other than 1, it does not seem that the model is accounting for this. In this case, this is a limitation of the approach, and it should be addressed in the text.

   Yes, the amount of firewood exported from a site depends on the number of campers leaving the site. We assume a linear relationship between the number of people going from patches $i$ to $j$, and the amount of wood from patches $i$ to $j$. However, detection of forest pests is often late, it indicates the already existing pest populations, and can significantly under-estimate infested tree population. For these reasons, no data is available regarding the proportions of infested wood carried by recreational travellers leaving sites, so we only assume that the total amount of infested wood leaving a site is dependent on the number of infested trees at that site.

   *We simplified the model by assuming that each infested patch provides similar propagule pressure to recreational travellers leaving the infested site. This assumption was made because no data about the actual proportions of infested wood carried by recreational travellers leaving the infested sites were available.*

3. How does $\phi$ model density dependence? What sort of density dependence is it accounting for? It makes sense to me if $\phi$ takes into account that there needs to be a sufficient number of pests attacking susceptible trees to overwhelm tree defenses and successfully infest the tree. If this is the sort of density dependence that $\phi$ is modeling then the argument should be $(I_i+B_i-I_a)$ instead of $(I_i-I_a)$, since infested firewood is also contributing pests(in exactly the same way as infested trees).

   In our case of tree pests which may be carried in firewood (like Emerald Ash Borer), the density dependence is not critical because a small number of larvae can establish a new viable population (which was demonstrated by a catastrophic expansion of emerald ash borer outbreak though eastern USA and Canada).

However the already established population on a tree has much higher capacity to spread at a given site than the larvae coming with imported firewood. We have made a simplifying assumption that the force of infection that one unit of firewood contributes is significantly smaller than one infested tree, and so the contribution of Bi in that term can be ignored. Assuming that they are equal in contribution, does make some small changes in specific threshold values, but does not qualitatively affect the results.

4. One thing that seems important in figure 2 is that increasing $C_e$ delays the initial peak in the infection. Increasing U does not have the same effect, probably due to a lag, since increasing $U$ must result in $L$ increasing before importation slows down.The shift in the initial peak in $I$ as $C_e$ increases is probably what leads to the delay in the increase in $L$ (with increasing $C_e$) in figure 2d. Increasing $C_e$ is probably not decreasing $L$ directly. It is just delaying its increase by delaying the peak in I.

   Yes, that is a good observation. We have amended the text to reflect this:
   *Increasing $C_e$ decreases the number of infested trees, the delays the peak of the outbreak (Fig 2b,c). The delay in the peak of the outbreak also appears to cause the lag in $L(t)$ (Fig 2d).*

5. Can you explain why increasing U would have a detrimental effect (increasing T) for some parameter values (Figs 4-6)? It seems like there should be some deeper explanation in terms of the dynamics of the infection (what would figure 2e-h look like in these cases?).It seems like at least some attempt should be made to explain this beyond just noting that it happens.This sort of understanding would add value to the paper.

   Figure 1 shows the timeseries for the point $(A, d) = (0.00126, 0.103)$. It seems that in these cases, the color scheme is misleading. The value of $dT(t)/dU$ at its largest, is still very close to zero (about $10^{-3}$). While the colorbar in the original figures did show this in the scale, the diverging color gradient was centered around zero. If we zoom in on the final 20 years in Figure 1e, we can still see a clear, albeit very small, increase in total infestation over 20 years, as we increase $U$(Figure 3). In panel f), we plot the number of patches where $I(t) \geq 1$ over time. It appears that since increasing $U$ (indirectly) slows transmission between patches, the pest is able to spend more time in the network, and therefore infect slightly more. We have changed the color scheme in the marginal return figures, and the language in the text to reflect this better.
   The timeseries of a point with good marginal return, such as $(A, d) = 0.00093, 0.038$ (Figure 2), looks significantly different.

In this parameter regime, reducing the initial peak also reduces the size of a 2nd outbreak, which eventually disappears if $U > 1$. We have added two of the timeseries to the SI appendix, and the following explanation to the text: *We see good marginal return in parameter regimes where few transport strategists (high $L(t)$) would reduce the reproductive ratio of the infection below 1. For instance, at the point $(A, d) = (0.00126, 0.103)$ , increasing $U$ is able to delay and eventually prevent a second wave, which decreases the total number of infected trees significantly (SI Fig 1). If the transmission rates $A, d$ are high enough that even with no transport strategists, we get a second wave of infection, the effect of increasing $U$ can be slightly detrimental (SI Fig 2). Panel f) of the aforementioned Figures 1,2 plots the number of patches where $I \geq 1$ over time. We see that in the case where $U$ is slightly detrimental ($\frac{dT(t)}{dU} > 0$), the infection persists longer and in more patches for larger $U$ (Figure 1f). The case where $\frac{dT(t)}{dU} << 0$, Figure 2f, shows that the infestation is not able to spread to as many patches and is usually eradicated more quickly.*

6. The authors did not satisfactorily address my comment regarding the focus on the marginal return on social incentives (basically dT(t)/dU). Focusing on this quantity may be obscuring more relevant dynamics. For example, if dT/dU is positive the infection peak may occur earlier but result in less infection when integrated over time. This could invalidate the statement starting in line 500. Even if dT/dU is positive, there may be more important beneficial effects of increasing U. Addressing my comment 5 may also help to address comment 6.

   It is true that using $\frac{dT(t)}{dU}$, as a first order approximation, could be obscuring some relevant dynamics. However, it is a useful way to get some insight out of the high dimensional data generated in our sensitivity analysis. If we understand your example correctly, it is one of the reasons we show $\frac{dT(t)}{dU}$ for at least a few different times $t$. For instance, in Figure 1, if $t = 5$, then we see that indeed $\frac{dT(t)}{dU} > 0$. If we zoom in on the end of the outbreak in Figure 1, then we see $t \geq 15$ then $\frac{dT(t)}{dU} < 0$, as shown in Figure 3. In Figure 4 in the main text, we plot $\frac{dT(t)}{dU}$ with respect to $t$. In this figure we see a situation similar to your example. For large $f$, $\frac{dT(t)}{dU}$ is closer to zero for $t < 10$, and then drops as we consider longer time horizons.

7. The authors did not satisfactorily address my comment regarding parameter choices and their effect on making specific claims/predictions. They addressed it by stating that their 'goal was not to provide predictions but

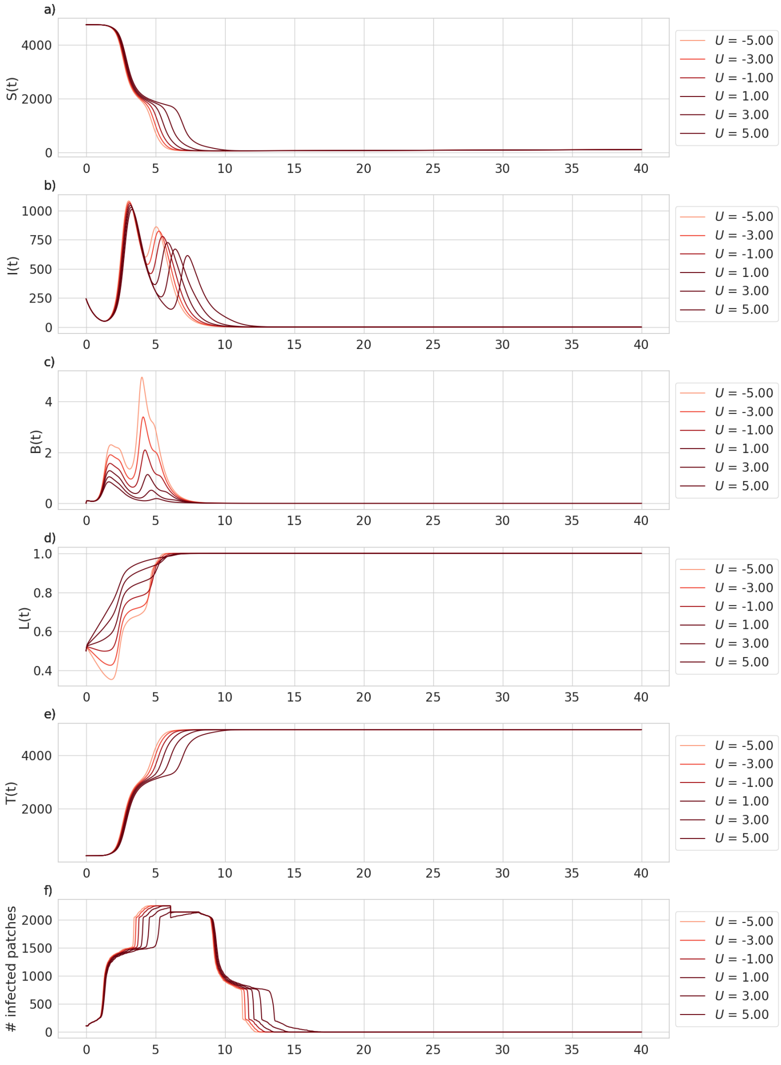


Figure 1: Time evolution of model variables for various values of $U$ where $(A, d) = (0.00126, 0.103)$.

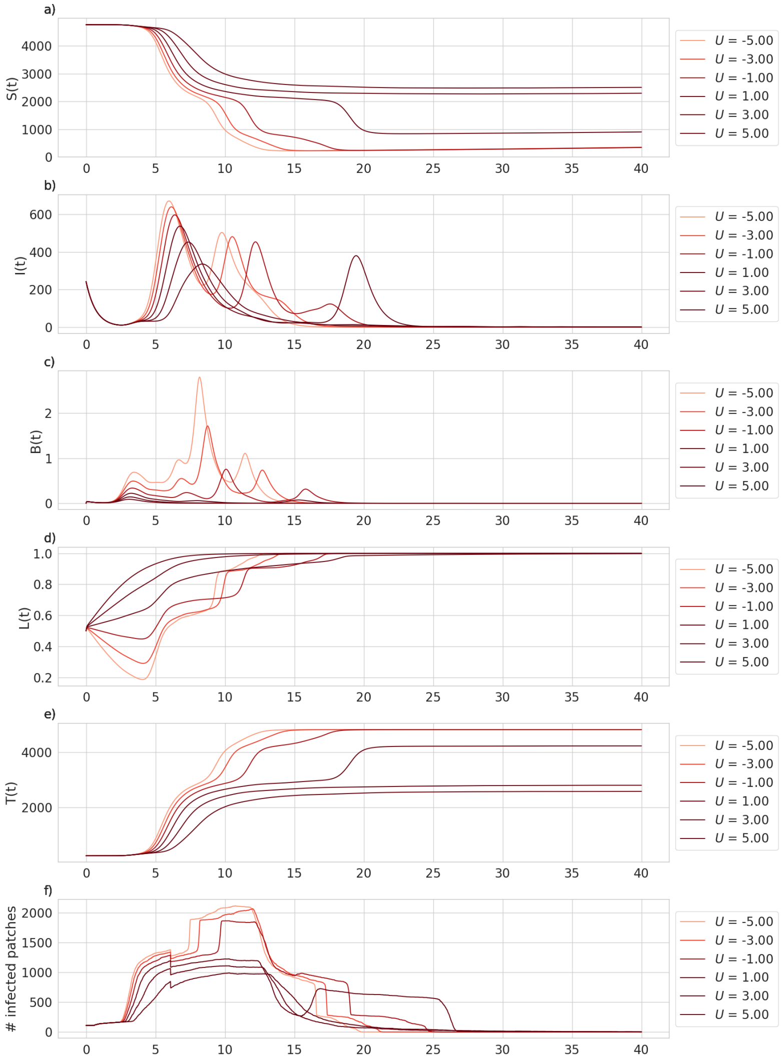


Figure 2: Time evolution of model variables for various values of $U$ where $(A, d) = (0.0009, 0.038)$.

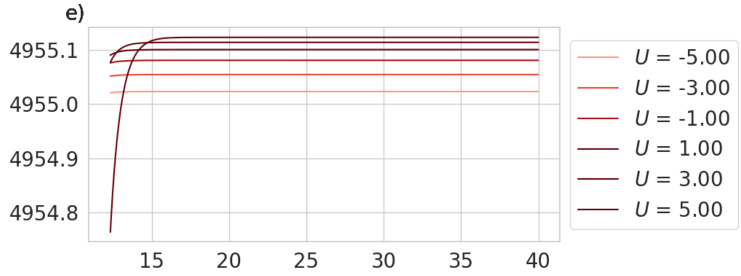


Figure 3: $T(t)$ for various values of $U$ where $(A, d) = (0.00126, 0.103)$, and $13 \leq t \leq 40$

to find insight into the behavior of these systems on networks'. However, their conclusions seem to imply some specific management recommendations (or at least I am afraid that they will be interpreted that way).Their conclusions are valid only if their parameter values are, and they have not justified their parameter choices. Here are some of the specific conclusions(paraphrased):

- Checkpoints are unlikely to be effective
- The total rate of interception to halve the infestation is 30%
- It is unlikely that quarantine measures could be effective
- At least 25% of firewood transports need to be intercepted to be effective

We have tried to rephrase the conclusion section to ensure that we are communicating them as outcomes of our particular model assumptions, rather than broad management recommendations.

1. Line 4: replace "on" with "for" or "to". Addressed.
2. Line 129-131. This sentence is not clear. It does not even seem necessary, so I recommend removing it. Removed.
3. Line 148. As defined, the function is not a "shifted" sigmoid. Remove "shifted". Removed.
4. Line 161. Remove "in". Removed.
5. Line 169. Replace "importation"with "exportation". Replaced.
6. Near line 183. The equation for $P_t$ should have $(0.5 - L_i)$ instead of $(L_i - 0.5)$. Addressed.
7. Near line 226. You misinterpreted my comment. The semicolon should go in the subscript for $\Sigma$. That is, it should read $i \neq s \neq t; t \in G$. Addressed.

8. Line 228. It should be stated that the weights are the $P_{ij}$s.
9. Lines 226-228. Be specific about how $g_{st}$ and $g_{st}(i)$ depend on the $P_{ij}$. (Write the equations)

   > We have addressed the above two points with the following addition to the text: *In our camper travel network, higher weights denote more frequent trips, so for the purposes of determining the betweenness centrality, the weight of each edge* $(i, j)$ *is* $max_{i,j}(P_{ij}) + 1 - P_{ij}$.

10. Equations 11-12. Replace epsilon with gamma. Addressed.
11. Lines 242-245. This sentence is unnecessary (obvious/redundant). Addressed.
12. Lines 254-256. This sentence is unnecessary(redundant). Addressed.
13. Line 378. Remove "is". Addressed.
14. Line 449 needs a verb. Addressed.
15. Line 449. "invasion speed" usually refers to spatial spread rate. That is not what figure 7 shows. Maybe just use "total infestation". Addressed.
16. Line 489. "is" should be "are". Addressed.
